# APP controls the formation of PI(3,5)P_2_ vesicles through its binding of the PIKfyve complex

**DOI:** 10.1007/s00018-015-1993-0

**Published:** 2015-07-28

**Authors:** Heather Currinn, Benjamin Guscott, Zita Balklava, Alice Rothnie, Thomas Wassmer

**Affiliations:** School of Life and Health Sciences, Aston University, Aston Triangle, Birmingham, B4 7ET UK

**Keywords:** Endosomal sorting, ML1Nx2, TRPML1, Mucolipin-1, Neurodegeneration

## Abstract

Phosphoinositides are signalling lipids that are crucial for major signalling events as well as established regulators of membrane trafficking. Control of endosomal sorting and endosomal homeostasis requires phosphatidylinositol-3-phosphate (PI(3)P) and phosphatidylinositol-3,5-bisphosphate (PI(3,5)P_2_), the latter a lipid of low abundance but significant physiological relevance. PI(3,5)P_2_ is formed by phosphorylation of PI(3)P by the PIKfyve complex which is crucial for maintaining endosomal homeostasis. Interestingly, loss of PIKfyve function results in dramatic neurodegeneration. Despite the significance of PIKfyve, its regulation is still poorly understood. Here we show that the Amyloid Precursor Protein (APP), a central molecule in Alzheimer’s disease, associates with the PIKfyve complex (consisting of Vac14, PIKfyve and Fig4) and that the APP intracellular domain directly binds purified Vac14. We also show that the closely related APP paralogues, APLP1 and 2 associate with the PIKfyve complex. Whether APP family proteins can additionally form direct protein–protein interaction with PIKfyve or Fig4 remains to be explored. We show that APP binding to the PIKfyve complex drives formation of PI(3,5)P_2_ positive vesicles and that APP gene family members are required for supporting PIKfyve function. Interestingly, the PIKfyve complex is required for APP trafficking, suggesting a feedback loop in which APP, by binding to and stimulating PI(3,5)P_2_ vesicle formation may control its own trafficking. These data suggest that altered APP processing, as observed in Alzheimer’s disease, may disrupt PI(3,5)P_2_ metabolism, endosomal sorting and homeostasis with important implications for our understanding of the mechanism of neurodegeneration in Alzheimer’s disease.

**Electronic supplementary material:**

The online version of this article (doi:10.1007/s00018-015-1993-0) contains supplementary material, which is available to authorized users.

## Introduction

In eukaryotes, the endosomal system plays a pivotal role for the sorting of endocytosed molecules and establishing which are to be reused and which ones are committed to destruction. The organisation of the endosomal system reflects this purpose. Endocytosed material is sorted in early endosomes for either recycling to the plasma membrane or retrograde transport to the trans-Golgi-network (TGN) (reviewed in [1]). As endosomes mature they acquire an increasing number of intraluminal vesicles into which cargoes destined for lysosomal degradation are sorted [2]. Ultimately, late endosomes undergo fusion with lysosomes, leading to the proteolytic degradation of transmembrane proteins contained within intraluminal vesicles as well as soluble protein contained in the fluid phase late endosomes.

Endosomal sorting is crucially underpinned by phosphoinositides. The signature lipid of the endosomal system is phosphatidylinositol-3-phosphate (PI(3)P) which is able to recruit a large number of PI(3)P binding proteins onto endosomes [3]. Binding of PI(3)P is enabled by a number of binding domains, well-characterised examples are PX- and FYVE-domains [4–6]. The significance of PI(3)P is apparent when its formation is inhibited by Wortmannin which disrupts numerous endosomal sorting processes [7].

Another endosomal phosphoinositide, PI(3,5)P_2_, has been far less studied, certainly in part because inhibitors of its formation have only recently become available. Furthermore, it is one of the least abundant phosphoinositides, considerably complicating its biochemical detection. PI(3,5)P_2_ is produced by phosphorylation of PI(3)P at the 5-position by the PIKfyve complex [8–10]. The PIKfyve complex consists in mammals of three subunits, PIKfvye (also known as Fab1), Vac14 (ArPIKfyve) and Fig4 (Sac3) [9]. The PIKfyve subunit contains the kinase domain, Vac14 acts as a scaffold for the complex, while Fig4 has an interesting dual role as a necessary activator of PIKfyve but can also function as a PI(3,5)P_2_ specific 5-phosphatase [8, 9, 11–13]. Recently it has been shown that the PIKfyve complex is the only source in mammalian cells for producing PI(3,5)P_2_ [14].

The most prominent and best established phenotype of PIKfyve dysfunction is the accumulation of aberrant vacuoles in the cytoplasm that stem from the endosomal system [15, 16]. The occurrence of these vacuoles has been established when a kinase-dead PIKfyve mutant was expressed, by RNAi suppression of PIKfyve, genetic ablation of PIKfyve complex members or pharmacological inhibition [15–18]. In every instance pronounced vacuolation indicated PIKfyve dysfunction. PIKfyve suppression also led to defective endosome-to-TGN transport of a number of cargoes [16, 19, 20]. How does PIKfyve function lead to vacuole formation? It was shown that PI(3,5)P_2_ is able to bind to and activate the TRPML-1 channel (also known as mucolipin 1). Inactivation of TRPML-1 replicates the vacuolation phenotype observed upon loss of PIKfyve, suggesting that the PIKfyve/TRPML-1 interplay is crucial for endosomal homeostasis [19].

When analysing the consequences of PIKfyve dysfunction, there is striking evidence that PIKfyve function is crucial for neuronal integrity [14, 17, 21]. In mouse models loss of function of any of its subunits by mutation or knock-out resulted in serious neurodegeneration and lethality shortly after birth [14, 17, 21]. Mutations in Fig4 have also been shown to lead to Charcot–Marie–Tooth syndrome and amyotrophic lateral sclerosis, both neurodegenerative disorders [21, 22].

How PIKfyve complex activity is controlled in metazoa currently remains entirely unclear. However, in a recent study, we identified the Amyloid Precursor Protein (APP) as a putative, novel interaction partner of the PIKfyve complex (Balklava et al., in press), raising the possibility that APP and the PIKfyve complex may have a shared function.

APP is a molecule known to be of central importance in Alzheimer’s disease, a progressive, neurodegenerative disease that results in debilitating incapacity of patients and is ultimately fatal. Alzheimer’s disease occurs in a sporadic form (also described as late onset Alzheimer’s disease) of unclear cause, while the familial form (known as early onset) is caused by mutations either in the Amyloid Precursor Protein (APP) gene or the APP cleaving gamma-secretase complex [23, 24].

A major step was the realisation that APP cleavage by the gamma-secretase, if preceded by so-called beta-secretase (BACE1) cleavage of APP results in a small, aggregation-prone peptide known as beta amyloid. This peptide can be found as a principle constituent of so-called ‘senile’ plaques that can be observed in patient brain sections. Both lines of evidence suggest that APP cleavage is a central event in Alzheimer’s disease [25].

Extensive work has been carried out to better understand APP processing by beta and gamma secretases and the pathophysiological consequences of beta-amyloid production. Much less effort has been dedicated to understanding the physiological role of APP (reviewed in [26]). APP has been shown to be enriched in synapses and is known to control synaptic transmission and synapse formation. However, the molecular mechanisms are not clear [26].

Further complicating the question concerning APP function is that besides APP two closely related APP paralogues, APLP1 and APLP2, exist in mammals. While APLP1 expression appears to be restricted to the brain, APP and APLP2 are ubiquitously expressed. Genetic analysis of the APP gene family strongly suggested that there is a large functional overlap between the different members of the APP gene family [27]. For example knock-out mice deficient for either APP or APLP2 have very subtle defects while double knock-out is peri-natally lethal for the vast majority of animals [27].

APP as a single spanning transmembrane protein is produced in the ER and traffics between the Golgi complex, the plasma membrane and endosomes (reviewed in [28]). Its trafficking has been studied in considerable detail. APP is sorted in the TGN by Adaptor protein complex 4 [29]. Endocytosis of APP is organised by AP-2, while endosomal sorting of APP is mediated by the retromer complex [30, 31] which enables endosome-to-TGN transport, and, depending on subunit composition, recycling to the plasma membrane [32–34].

Our recent work aimed to establish the interactome of the intracellular domain of APP to elucidate its function. Surprisingly, we found that all three subunits of the PIKfyve complex (e.g., PIKfyve, Vac14 and Fig4) associated with the intracellular domain of APP (Balklava et al., in press). When studying the interplay using *C. elegans* genetics, it became clear that APP functionally interacts with the PIKfyve complex, suggesting that APP and PIKfyve function in the same pathway. However, the mechanism of this remained unclear.

Here we show that APP directly binds Vac14 of the PIKfyve complex. We demonstrate that overexpression of APP or its intracellular domain AICD stimulates the formation of PI(3,5)P_2_ positive vesicles in a PIKfyve-dependent manner. Conversely, RNAi mediated suppression of APP and APLP2 decreased the number of PI(3,5)P_2_ vesicles, suggesting that APP gene family members regulate endosomal sorting via PIKfyve. We also show that suppression of APP gene family members increased incidence of vacuoles and cellular susceptibility to vacuole formation when PIKfyve function becomes compromised. Finally, we show that PIKfyve activity is required for successful sorting of APP at endosomal membranes, establishing a complex and reciprocal relationship between APP and PIKfyve with interesting implications for our understanding of Alzheimer’s disease.

## Materials and methods

### Antibodies

APP beta-amyloid (sc-53822), LampI (sc-20011), Vac14 (sc-271831), PIKfyve (sc-100408) (all from Santa Cruz Biotechnology). APLP2 (ab140624) (Abcam). EEA1 (610457) (BD Biosciences). MBP (2396S), Anti-mouse IgG, HRP-linked (7076), Anti-rabbit IgG, HRP-linked (7074), Anti-rabbit IgG Fab2 Alexa Four 555 (44135), Anti-mouse IgG Fab2 Alexa Four 555 (44095) (All from Cell Signalling Technology).

### Plasmids

pEGFP-n1-APP was created by PCR amplification of APP695 from cDNA extracted from HeLa cells and cloned into pEGFP-n1 (Clontech), followed by subcloning into pmCherry-n1 and pYFP-n1 using *Nhe*I and *Sal*I. The intracellular domain was removed by PCR amplification of APP and cloning into pYFP-n1 using *Nhe*I and *Sal*I. AICD and the various AICD truncation mutations were PCR amplified using pEGFP-n1-APP as template and cloned into pEGFP-n1 or pYFP-n1. For bacterial expression the various truncations were PCR amplified and cloned into a pET28 derived plasmid described in [35] using *Bam*HI and *Xho*I. pEGFP-c3-2xML1N was kindly provided by Dr. H. Xu (University of Michigan) [36] and was PCR cloned into pmCherry-c1 using *Xho*I and *Bam*HI. The expression plasmid for production of His-tagged Vac14 was kindly provided by Dr. L. Weisman (University of Michigan). Expression plasmids for the APLP1 and 2 intracellular domains as well as AICD mutations in the YENPTY motif were synthesised by GeneArt (Life Technologies) with codon-optimisation for *E. coli* and subcloned into pET28-MBP-TEV using *Xho*I/*Bam*HI for bacterial expression.

### Proteo-liposome recruitments for analysing the APP/PIKfyve complex interaction

Recombinant 6xHis MBP tagged cytoplasmic receptor tails were expressed in *E. coli* (Bl21DE3) and purified as described in [35, 37]. Recruitments were carried out as described in [35, 37] with the following modification: The proteo-liposomes were separated from the cytosol or purified Vac14 using a sucrose cushion (2 ml 60 % sucrose in RB and 8 ml 5 % sucrose in RB) in a Beckman coulter optima L-100k ultracentrifuge (SW40 rotor) at 38,000 rpm for 90 min at 4 °C. The interface between the two sucrose phases containing the proteo-liposomes was removed and re-suspended in 11 ml of Recruitment Buffer (RB) and pelleted in a Beckman coulter optima L-100k ultracentrifuge (SW40 rotor) at 38,000 rpm for 1 h at 4 °C. The supernatant was discarded and the pellet re-suspended in 100 µl of 2× Laemmli buffer. The samples were analysed by SDS-PAGE and western blotting for interacting proteins.

### Purification of recombinant Vac14

Recombinant, His-tagged Vac14 was expressed at 25 °C for approximately 18 h and purified using Ni-NTA (Pierce) according to the manufacturers protocol.

### Maltose binding protein pull-downs for testing the interaction of recombinant Vac14 with AICD

20 µg of purified recombinant, MBP-AICD, MBP-Tr1, MBP-Tr2, MBP-Tr3, MBP-Tr4 and MBP were incubated with 10 µg of purified His-Vac14 for 1 h rocking on ice in 200 µl PD-buffer (25 mM HEPES pH 7.0, 50 mM KCl, 1 mM MgCl_2_) followed by incubation with 20 µl of amylose resin beads (New England Biolabs) equilibrated in PD-buffer. Samples were incubated rocking on ice for 1 h and the amylose beads spun down using centrifugation (1000 rpm, 1 min) and washed 5 times using PD-buffer. Proteins were eluted by incubating the beads with 50 µl of PD-buffer containing 20 mM maltose for 10 min on ice, followed by SDS-PAGE and western blotting.

### Transfections

HeLa cells were seeded onto glass coverslips in a 24-well plate at a density of 100,000 cells per well and incubated at 37 °C with 5 % CO_2_ overnight. The following day the cells were co-transfected with a combination of the plasmids indicated using Lipofectamine 2000 (Invitrogen) according to the manufacturer’s protocol. Cells were fixed and mounted the following day or selected with G418 for the isolation of a stably transfected population.

### Fixed cell imaging and immunostaining

HeLa cells were fixed for 20 min in 4 % paraformaldehyde depolymerised in PBS followed by two washes in PBS. Cells were permeabilised using 0.1 % Triton X-100 in PBS for 4 min followed by two washes in PBS and blocking using 2 % bovine serum albumin (BSA) in PBS for at least 15 min. Cells were stained by use of a primary antibody (diluted in 2 % BSA, incubation for 1 h at RT) followed by three washes in PBS and incubation with the secondary antibody (diluted 1:500 in 2 % BSA, incubation for 1 h, RT. Finally, cells were washed thrice using PBS and mounted using Mowiol. Samples were imaged on an Leica SP5 TCS II MP confocal microscope with a 63× oil immersion lense.

### Quantification of APP-GFP localisation in relation to EEA1, LampI and GM130

Image analysis was performed using ImageJ following [38] for analysis of ciMPR localisation with the following modification: Briefly, EEA1, LampI or GM130 staining was used to define a mask by applying a manual threshold, defining organelles positive and negative for the respective marker. Then the APP-GFP label inside the mask was measured as well as the total cellular APP-GFP label, using the ‘ImageJ’s ‘Histogram’’ function. APP-GFP label inside the mask was calculated as a percentage of total APP-GFP label.

Live cell imaging for the observation of APP-GFP and mCherry-ML1Nx2 was carried out as describe in [39].

### Quantification of ML1N positive vesicles

Images were imported into ImageJ and maximum projections created from the Z-stacks. Each cell to be quantified was individually selected. mCherry ML1Nx2 positive structures were analysed using the MosaicSuite for ImageJ [40]. This allows automatic image segmentation, automatic detection of structures such as labelled vesicles and quantification of the number of detected objects as well as object intensity and object shape [40]. The advantage of this methodology is that the algorithm makes no assumptions about the shape of the objects to be detected [40]. Furthermore, it does not require any manual initialisation of the segmentation process, precluding the introduction of bias by the observer. The following parameters were used: Background subtraction: 10. Regularisation: 0.1. Minimum object intensity: 0.3 (for overexpression experiments) and 0.15 (for RNAi experiments). The average number of mCherry-ML1Nx2 structures per cell was measured using the MosaicSuite for each condition together with the average intensity of these structures. Statistical significance of the data was analysed using a one-way ANOVA (*α* = 0.05) with a Tukey’s post hoc test in GraphPad Prism 6.

### RNAi suppression

HeLa cells were seeded at a density of 50,000 cells/well in a 24 well plate. The following day, prior to transfection, the medium was exchanged. The transfection mix containing 100 µl Optimem, 12 pmol of RNAi duplex and 3 µl of Interferin (PolyPlus) was prepared, incubated for 20 min at RT and added to cells, resulting in a final RNAi concentration of 20 nM. Cells were fixed for imaging or lysed for Western experiments 3 days after transfection.

### Vacuole quantification

3 days post-RNAi transfection living cells were imaged using a Nikon Eclipse TS100 microscope with a 40× objective. A minimum of 25 cells per image were scored manually for the presence of vacuoles as previously established [16].

### PIKfyve inhibition using YM201636 and Apilimod

YM201636 was purchased from Abcam (ab141370). Apilimod was purchased from USBiolgical (002800). Both YM201636 and Apilimod were applied at the concentrations and for the times indicated in the figure legends. It was noted that the YM201636 PIKfyve inhibitor easily precipitated in the presence of various transfection reagents. Therefore, cells were washed at least three times with complete medium before YM201636 was applied.

### Statistical treatment

As in all experiments multiple samples were compared we utilised one-way ANOVA (*α* = 0.05) tests in GraphPad Prism 6 with Tukey’s or Dunett’s post hoc tests.

## Results

### APP interacts with the Vac14 subunit of the PIKfyve complex

Until recently the study of interaction partners of the intracellular domain of transmembrane receptors, particularly their association with coats and other trafficking regulators has been exceedingly difficult. The major contributing factor to this difficulty has been that coats and their regulators rely on membrane attachment as well as the binding to cytoplasmic domains of receptors [41]. In the absence of a membrane context, it can be extremely difficult to detect the binding of coats to receptors.

This limitation has been overcome with the creation of the proteo-liposome system, in which the cytoplasmic domains of transmembrane proteins is covalently coupled to preformed liposomes in a sterically defined manner [35, 37, 42]. This mimics the situation encountered in cells where the cytoplasmic receptor domain is presented with a membrane environment, allowing the efficient recruitment of coats and their regulators [35, 42]. In our recent study we utilised this powerful system for identifying novel interaction partners of the APP intracellular domain (known as AICD) using mass spectrometry (Balklava et al., in press).

**Fig. 1:**
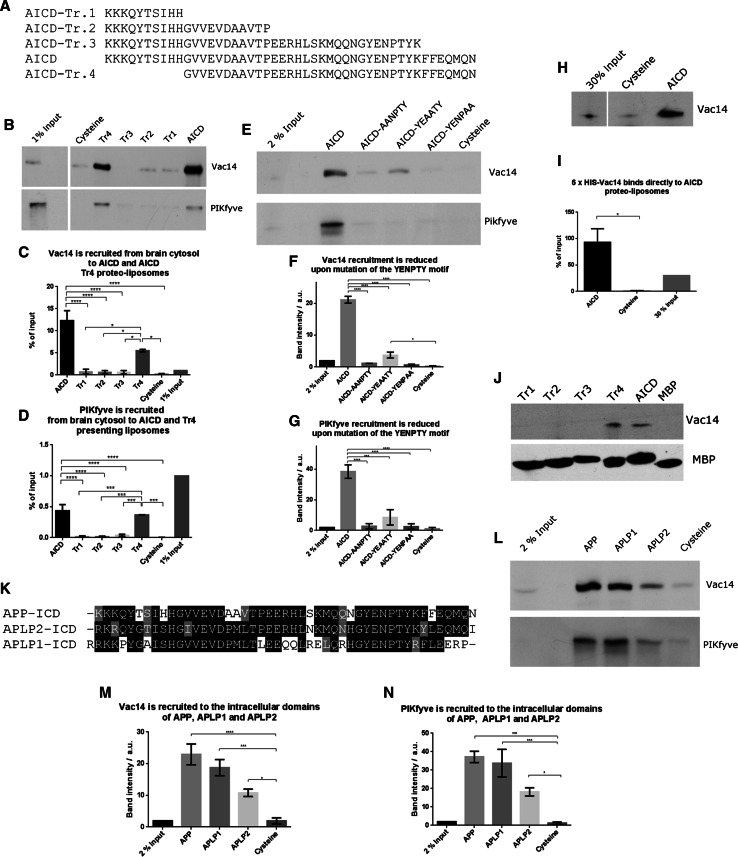
Mapping of the interaction between APP’s intracellular domain and the PIKfyve complex. **a** Sequences of the APP intracellular domain AICD and truncations utilised. **b** Proteo-liposomes displaying cysteine (negative control), AICD or truncations of AICD. AICD and the N-terminal AICD-Tr.4 recruited PIKfyve and Vac14 of the PIKfyve complex from pig brain cytosol while the negative control and all C-terminal AICD truncations failed to do so. **c**, **d** Quantification of Vac14 and PIKfyve bound to proteo-liposomes displaying AICD and AICD truncation mutants (*n* ≥ 3). Significant differences (ANOVA test followed by Tukey’s post hoc analysis with *α* = 0.05) are indicated. *p ≤ 0.05, **p ≤ 0.01, ***p ≤ 0.001, ****p ≤ 0.0001. *Error bars* are s.e.m. **e** Double-point mutations in the highly conserved YENPTY motif and their impact on AICD’s ability to interact with PIKfyve or Vac14. AICD mutations AANPTY and YENPAA fully abolished its binding to Vac14 while YEAATY strongly reduced the interaction. **f**, **g** Quantification of YENPTY mutants and their interaction with Vac14 and PIKfyve (ANOVA test followed by Tukey’s post hoc analysis with *α* = 0.05. *p ≤ 0.05, **p ≤ 0.01, ***p ≤ 0.001, ****p ≤ 0.0001. *Error bars* are s.e.m.). **h** AICD bound purified Vac14 in protein recruitments while the negative control, cysteine did not. **i** Quantification of binding of purified Vac14 to AICD proteo-liposomes. Analysed by Student’s *t* test, *p ≤ 0.05, *n* = 3. **j** MBP pull-downs showed that AICD and AICD-Tr.4 interact with purified Vac14 while the MBP (negative control) or AICD truncations 1–3 did not, confirming that AICD binds Vac14 directly. **k** Protein sequence alignment of the intracellular domains (ICD) of APP gene family members, highlighting the high degree of homology of the intracellular domains. **l** APLP1 and, to a lesser extent, APLP2 are both able to bind Vac14 and PIKfyve in proteo-liposome recruitments compared to a cysteine control. **m**, **n** Quantification of Vac14 and PIKfyve binding to APP, APLP1 and APLP2 (ANOVA test followed by Dunett’s post hoc analysis with *α* = 0.05. *p ≤ 0.05, **p ≤ 0.01, ***p ≤ 0.001, ****p ≤ 0.0001. *Error bars* are s.e.m., *n* = 3)

Here, we utilised this system to study the interaction between APP and the PIKfyve complex in detail. First we created three C-terminal truncations and one N-terminal deletion of the 47 amino acid long AICD and tested their potential for binding the PIKfyve complex using proteo-liposomes. Full length AICD and an N-terminal deletion removing 10 amino acids (called AICD-Tr.4) are both capable of binding both Vac14 and PIKfyve from pig brain cytosol (Fig. 1a–d). In contrast, any C-terminal truncation removing 37, 26 or 7 amino acids from AICD (AICD-Tr.1, 2 and 3, respectively), abolished binding of both Vac14 and PIKfyve. To explore the nature of the binding site in more detail, we turned our attention to the highly conserved YENPTY motif next to the deletion introduced in AICD-Tr.3. This motif is conserved in evolution from humans down to very simple metazoans such as the cnidarian *Nematostella*, arguing for an important and conserved function of this motif. We created three ‘double point’ mutations, exchanging two amino acids in full length AICD for two alanines, yielding the mutants: AICD-AANPTY, AICD-YEAATY, and AICD-YENPAA. We tested their binding capacity and found that any of the three mutations either abolished or strongly reduced binding of Vac14 and PIKfyve in proteo-liposome recruitments, demonstrating that this motif in addition to sequence elements identified using AICD-Tr.3 are necessary for APP binding of the PIKfyve complex (Fig. 1e–g). These data suggested that the PIKfyve complex binding site is located in close proximity to the C-terminus of AICD and spans the conserved YENPTY motif.

Next we tested the binding capability of bacterially expressed and purified Vac14 and found that it bound AICD on proteo-liposomes (Fig. 1h, i). We also tested the binding of purified Vac14 using classical pull-downs. We found that both AICD and Tr.4 were able to bring down Vac14, while Tr. 1–3 and the negative control MBP failed to bind Vac14, fully recapitulating the results obtained using proteo-liposome recruitments (Fig. 1j). These data suggest that APP forms a direct protein–protein interaction with the Vac14 subunit of the PIKfyve complex.

As the YENPTY sequence motif is also found in the APP related genes, APLP1 and APLP2 (alignment shown in Fig. 1k) we tested their ability to bind Vac14. We found that both APLP1 and APLP2 were able to bind Vac14 and PIKfyve (Fig. 1l–n), suggesting that all members of the mammalian APP gene family are likely to be PIKfyve complex interactors.

### APP or AICD overexpression stimulates the formation of ML1Nx2 positive vesicles

What is the functional significance of this interaction? Most previous work has focused on the roles that APP or the APP gene family may play in neurons. However, APP and APLP2 are expressed ubiquitously, while APLP1 expression appears to be restricted to neurons [43]. This clearly suggests that the APP gene family is likely to have functions not limited to the brain. The PIKfyve complex is also widely expressed [44]. To test for a generic APP function we choose the epithelium derived HeLa cell line, in which the function of PIKfyve has been thoroughly characterised [16, 20].

First we asked whether overexpression of APP or APP’s intracellular domain may alter production of PI(3,5)P_2_. To test this, we utilised the recently established PI(3,5)P_2_ specific probe ML1Nx2 [36]. Fusion of a tandem repeat of this lipid binding domain of the PI(3,5)P_2_ binder TRPML-1 to a fluorescent protein allows the detection of PI(3,5)P_2_ in a spatially and temporally defined manner which had not been previously possible [36]. This probe has also been used successfully in a study that established that PIKfyve function is required for AMPA receptor trafficking and synaptic depression, further validating it as a tool for analysing PI(3,5)P_2_ dynamics in vivo [45]. mCherry-ML1Nx2 was transfected into cells and analysed by confocal microscopy. Structures labelled by ML1Nx2 were analysed using the MosaicSuite segmentation tool of ImageJ, allowing unbiased, automated detection of ML1Nx2 positive structures and their intensity.

**Fig. 2:**
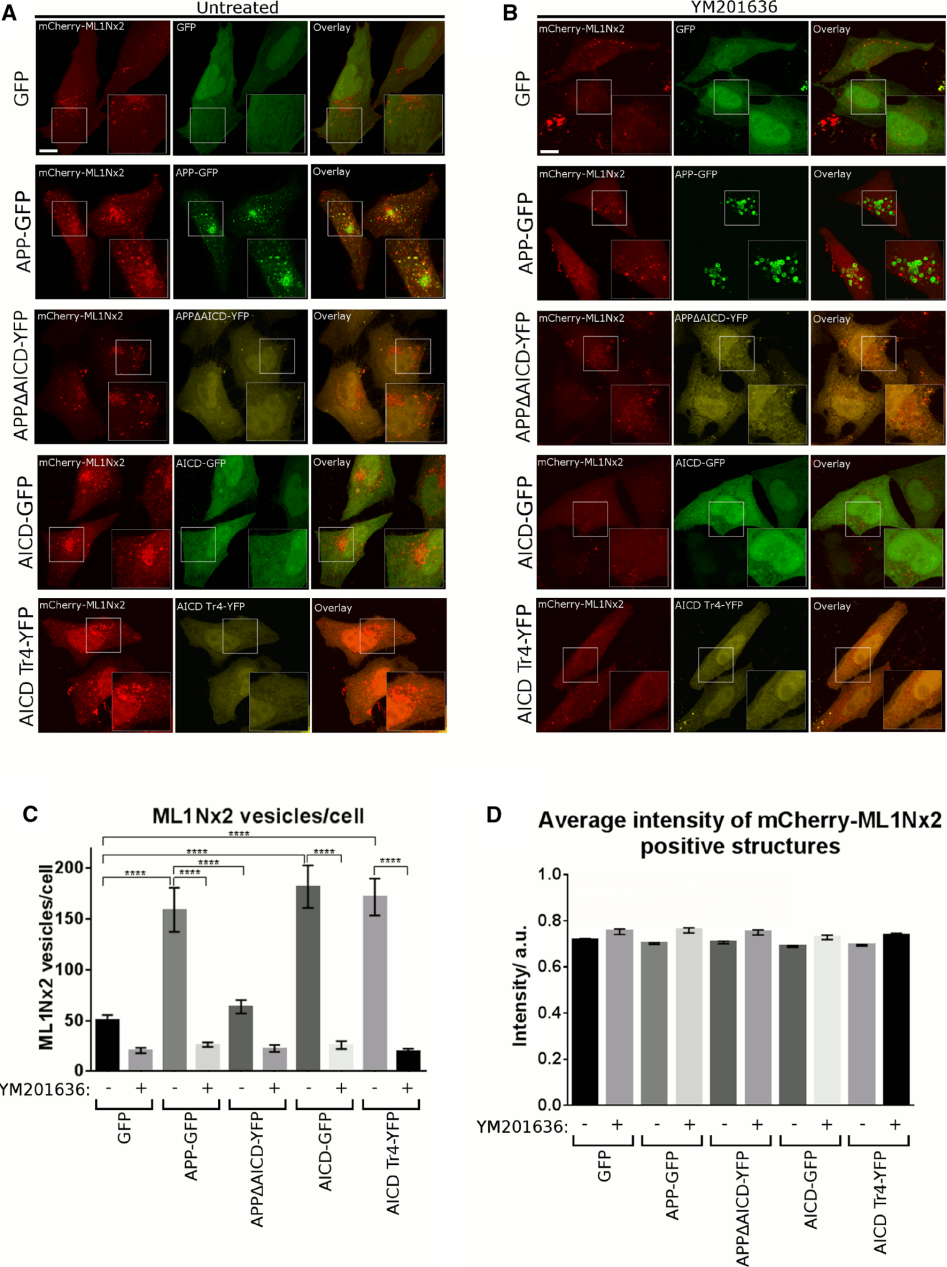
Overexpression of APP and AICD modulates PIKfyve function. **a**, **b** Co-expression of GFP-tagged proteins and the mCherry-labelled PI(3,5)P_2_ specific probe ML1Nx2 was used to analyse the impact of APP-derived constructs on PI(3,5)P_2_ positive structures in the absence (**a**) or presence of the PIKfyve inhibitor YM201636 (**b**). Expression of APP, AICD or the N-terminal AICD truncation mutant AICD-Tr.4 all increased the average number of ML1Nx2 positive vesicles. In contrast, APP lacking its intracellular domain (APPΔAICD) and C-terminal AICD truncations (Tr. 2 and 3) failed to increase the number of ML1Nx2 positive vesicles (Online Resource 1). **b** The incidence of ML1Nx2 positive vesicles could be nearly eliminated by PIKfyve inhibition (4 µM YM201636 for 4 h), demonstrating that formation of ML1Nx2 positive vesicles is indeed PIKfyve dependent. *Bar* 10 µm. **c** Automated quantification of the number of mCherry-ML1Nx2 structures of 25 cells pooled from three independent experiments for each condition were analysed (with 8–10 stacks acquired from each experiment) using image segmentation (MosaicSuite in ImageJ [40]). Significant differences (ANOVA test followed by Tukey’s post hoc analysis with *α* = 0.05) are indicated. ****p ≤ 0.0001. *Error bars* are s.e.m.). **d** Quantification of average ML1Nx2 intensity as analysed using MosaicSuite. APP, AICD and AICD-Tr.4 expression did not majorly affect the average intensity of mCherry-ML1Nx2 vesicles, suggesting that APP controls the number of ML1Nx2 positive vesicles rather than the PI(3,5)P_2_ amount per vesicle. *Error bars* are s.e.m.

Overexpression of APP-GFP led to a strong increase in the number of ML1Nx2 positive vesicles per cell compared to the expression of GFP as a negative control (Fig. 2a, c). By contrast, overexpression of APP lacking the intracellular domain (APPΔAICD) did not significantly alter the number of ML1Nx2 positive vesicles, suggesting that the intracellular domain of APP is required for stimulating the formation of ML1Nx2 positive vesicles (Fig. 2a, c). To confirm that the increase of ML1Nx2 positive vesicles upon overexpression of APP truly depends on PIKfyve activity we combined APP overexpression with pharmacological PIKfyve inhibition using YM201636 [18]. Upon PIKfyve inhibition the number of ML1Nx2 vesicles was drastically reduced, demonstrating that the APP-induced increase of ML1Nx2 positive vesicles is indeed dependent on PIKfyve activity (Fig. 2b, c). This is fully in line with the recent report of the Weisman lab that PIKfyve-dependent phosphorylation of PI(3)P is the only source for the production of PI(3,5)P_2_ in mammals [14]. Next we asked whether expression of the intracellular domain of APP, AICD, was also capable of stimulating the formation of ML1Nx2 positive vesicles. AICD, when released from APP by gamma-secretase cleavage, is a soluble, cytosolic molecule that loses its membrane attachment. We found that AICD-GFP expression strongly stimulated the formation of ML1Nx2 positive vesicles, mimicking the effect observed upon APP overexpression (Fig. 2a, c). When testing the truncation mutants we found that AICD-Tr.4 was able to stimulate the formation of ML1Nx2 positive vesicles to a similar extent as full length AICD (Fig. 2a, c), while AICD-Tr.2 and AICD-Tr.3 failed to do so (Online Resource 1). It is interesting to note that both AICD and AICD-Tr.4 are still able to stimulate formation of ML1Nx2 positive vesicles, despite neither of them being membrane attached by a transmembrane domain. Binding of the PIKfyve complex (as established in Fig. 1a–d) seems to be sufficient for the ability to provoke an increase in ML1Nx2 positive vesicles.

In summary, in all AICD mutants characterised so far, the ability to bind Vac14 perfectly correlated with their ability to stimulate the formation of ML1Nx2 positive vesicles upon overexpression. These data suggest that APP requires Vac14 binding to stimulate PIKfyve-dependent formation of ML1Nx2 positive vesicles.

To explore the nature of the ML1Nx2 vesicles induced by overexpression of APP we performed triple labelling using APP-GFP, mCherry-ML1Nx2 and staining for EEA1 or LampI. The large majority of APP/ML1Nx2 positive structures were also positive for EEA1, suggesting that these vesicles are early endosomal in nature (Online Resource 2).

We also wanted to test whether APP or AICD overexpression has the same effect in a neuronal cell line. APP-GFP or AICD-GFP were co-expressed together with mCherry-ML1Nx2 in the SH-SY5Y neuroblastoma line and compared to a GFP control (Online Resource 3). As in HeLa cells both APP and AICD expression increased the number of ML1Nx2 positive structures. Interestingly, in SH-SY5Y cells APP seemed more effective than AICD at increasing the number of ML1Nx2 vesicles.

These data show that in both neuronal and non-neuronal cell lines overexpression of APP or AICD increased the number of ML1Nx2 positive vesicles.

### Suppression of APP and/or APLP2 compromises PIKfyve-dependent processes

**Fig. 3:**
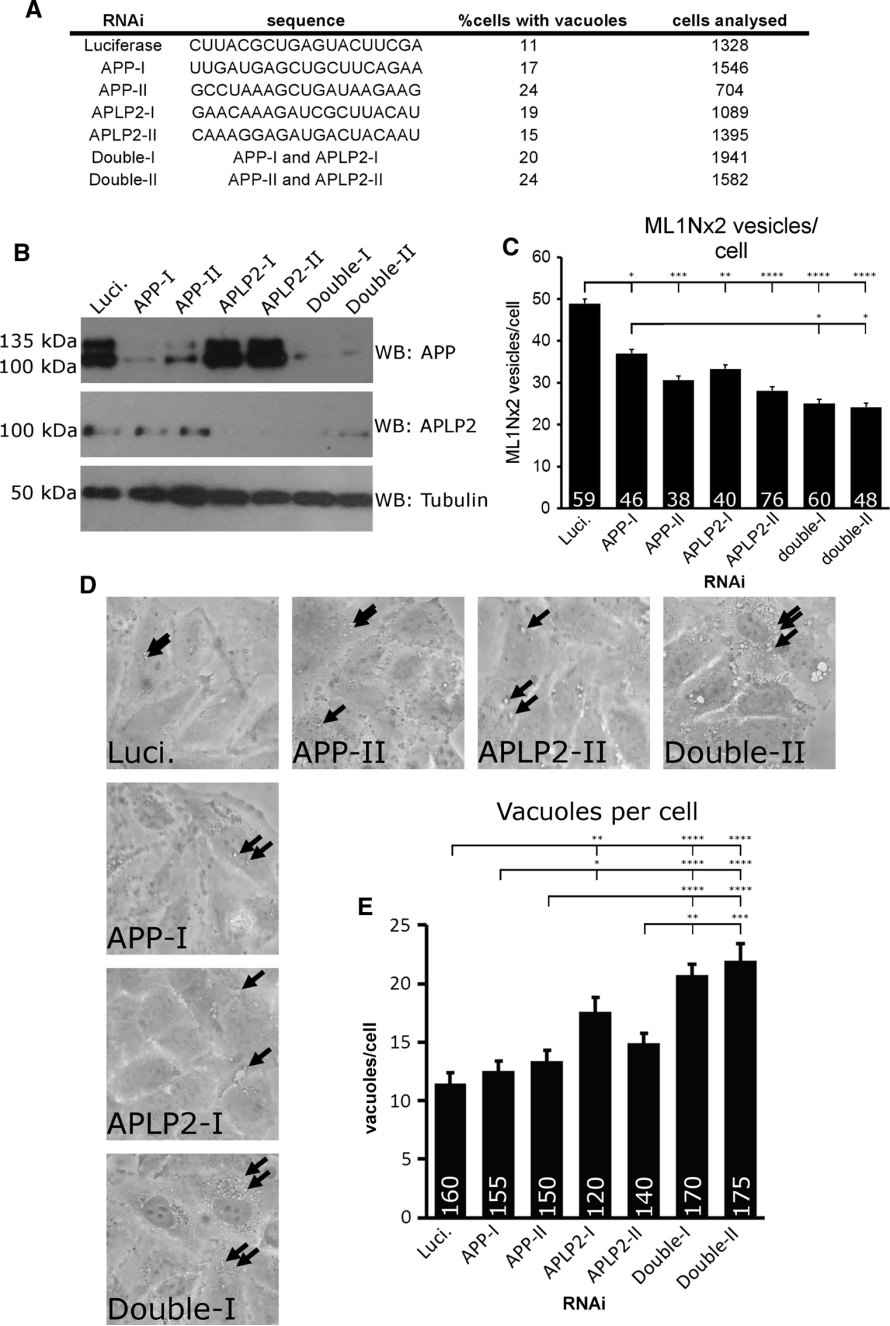
APP and APLP-2 suppression disrupts PIKfyve-dependent processes. **a** siRNA duplex sequences utilised and percent of HeLa cells in which vacuoles were observed after suppression of APP, APLP2 or double suppression. Incidence of vacuoles was increased upon single or joint suppression of both APP and APLP2. **b** Confirmation of successful knock-down of APP and APLP2 by western blotting. **c** Joint suppression of APP and APLP2 by RNAi and automated detection of ML1Nx2 positive vesicles. Single or joint suppression of APP or APLP2 resulted in a significant reduction of ML1Nx2 positive structures compared to the Luciferase negative control duplex (ANOVA test followed by Tukey’s post hoc analysis, *n* = 3). **d**, **e** RNAi suppression of APP and APLP2 combined with limited PIKfyve inhibition (1 µM, 45 min) and quantification of the number of apparent number of vacuoles. Under these conditions only few, small vacuoles (*arrows*) were detected in the Luciferase control, while suppression of APLP2 using duplex I and double suppression of both APP and APLP2 significantly increases vacuole number (quantified in **e**), suggesting that joint suppression of APP and APLP2 sensitises HeLa cells for PIKfyve inhibition. **c**, **e** Statistical test using ANOVA followed by Tukey’s post hoc analysis. *α* = 0.05, only significant differences are indicated, *p ≤ 0.05, **p ≤ 0.01, ***p ≤ 0.001, ****p ≤ 0.0001. Number of cells analysed per condition are indicated in each *bar* in the diagram. *Error bar*s are s.e.m., *n* = 3)

Are APP gene family members required for PIKfyve activity? We tested this question using RNAi mediated suppression of APP and the paralogue APLP2. Effective suppression of APP, APLP2 or double suppression was achieved with two different siRNA duplexes per gene (Fig. 3a, b). Automated detection of ML1Nx2 positive structures was used to test whether ML1Nx2 positive vesicles are affected by APP gene family suppression. Both single and double suppressions of APP and APLP2 led to a reduction of the number of ML1Nx2 positive vesicles (Fig. 3c). This showed that APP and APLP2 are required for PI(3,5)P_2_ vesicle formation, not entirely surprising given the high similarity of their intracellular domains and their potential to interact with the PIKfyve complex.

It is well established that loss of PIKfyve activity induced by expression of a kinase-dead version of PIKfyve, RNAi suppression, mutation of PIKfyve complex genes or pharmacological PIKfyve inhibition leads to a dramatic and consistent accumulation of vacuoles in cells, aberrant structures that were previously shown to be derived from the endosomal system [16, 17, 20, 46]. We analysed whether RNAi suppression of APP and/or APLP2 led to such vacuoles. Suppression of APP and/or APLP2 led to an increase of vacuole incidence compared to a control siRNA (Fig. 3a). It is worthwhile noting that occurrence of vacuolation is less pronounced with APP gene family knock-down than with PIKfyve RNAi [16], suggesting that APP family genes play an auxiliary rather than an essential role in preventing vacuolation.

This idea would suggest that suppression of APP and APLP2 will sensitise cells for PIKfyve inhibition. We tested this by inhibiting PIKfyve for a short period of time (45 min). In control RNAi treated cells this brief PIKfyve inhibition led to the formation of a number of small vacuoles. However, single suppression of APLP2 using RNAi duplex I or double suppression of APP and APLP2 significantly increased the incidence of vacuoles when PIKfyve was briefly inhibited (Fig. 3d, e). APP or APLP2 RNAi duplex II had no significant effect on vacuolation. These data show that double suppression of APP and APLP2 sensitises cells for reduced PIKfyve activity, leading to increased vacuolation. In this assay single suppressions had no statistically significant effect (APP duplexes) or variable effects in the case of APLP2.

### APP requires PIKfyve activity for its trafficking

**Fig. 4:**
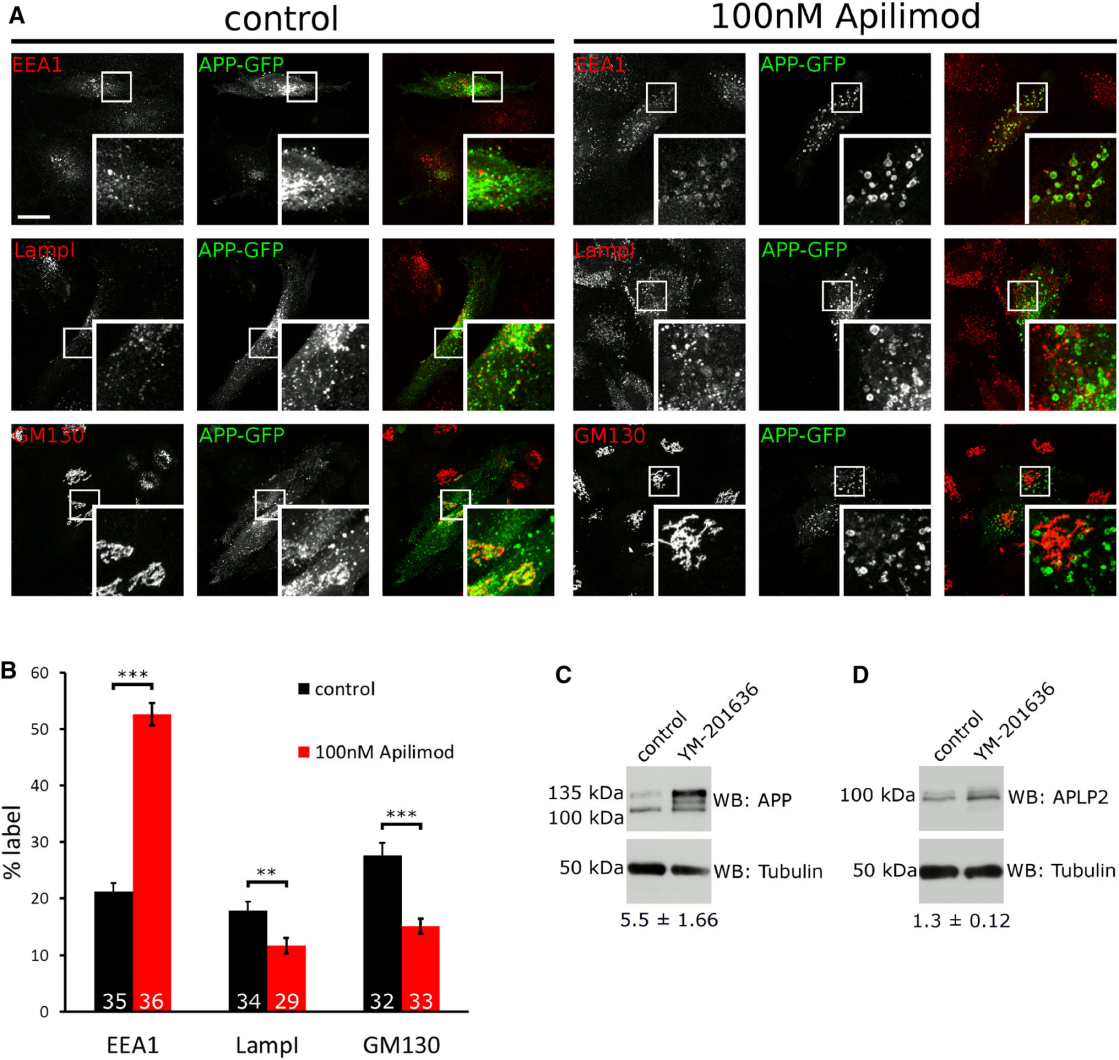
Inhibition of PIKfyve disrupts endosomal sorting of APP. **a** APP-GFP localisation in the absence or presence of the PIKfyve inhibitor Apilimod (100 nM, 4 h), co-labelled with early endosomal marker EEA1, late endosomal/lysosomal marker LampI and Golgi marker GM130. Upon PIKfyve inhibition, APP accumulated in EEA1 positive vacuoles, while little overlap with LampI was detected. PIKfyve inhibition also reduced APP label detectable in the Golgi area. **b** Quantification of APP-GFP label in EEA1, LampI and GM130 structures. EEA1, LampI or GM130 staining was used to define a mask and the percentage of cellular APP label contained inside the mask was measured in three independent experiments and expressed as a percentage of total APP-GFP label. PIKfyve inhibition led to a marked increase of APP in EEA1 positive structures, while APP label in LampI and GM130 positive structures was significantly decreased as tested using unpaired Student’s *t* test (**p < 0.01, ***p < 0.001, *error bars* are s.e.m.). Total number of cells analysed per condition is indicated in each *bar* in the diagram. Between 10 and 12 stacks were collected from three independent experiments and pooled. **c** APP accumulated in cells upon overnight inhibition of PIKfyve using 4 µM YM201636 as analysed by western blotting. The total of all APP species was increased approximately fivefold (*n* = 6). Particularly the 135 kDa form was affected by PIKfyve inhibition. **d** No major change was detected for APLP2

What is the purpose of APP binding to PIKfyve and stimulating the formation of ML1Nx2 positive vesicles? We noticed that upon PIKfyve inhibition using YM201636 a marked redistribution of APP-GFP occurred; instead of localising to small vesicles throughout the cell, APP became trapped in large, vacuolar structures (Fig. 2b). We analysed APP-GFP localisation in more detail by inhibiting PIKfyve using YM201636 and the recently established Apilimod [47] in HeLa cells. APP-GFP accumulation in vacuoles was dose dependent for both inhibitors with mild effects observed with as little as 3 nM Apilimod (Online Resource 4) and 100 nM YM201636 (Online Resource 5) for 4 h. APP-GFP accumulation in vacuoles became progressively worse over a 4 h time course with both inhibitors (1 µM YM201636 or 30 nM Apilimod) (Online Resources 6 and 7). Next we analysed in what organelles APP-GFP accumulated. We analysed the early endosomal marker EEA1, late endosomal and lysosomal marker LampI as well as the Golgi marker GM130. In control cells APP could be detected in EEA1, LampI and GM130 compartments, consistent with its established trafficking pattern [29, 48]. However, when PIKfyve was inhibited using Apilimod we found a marked redistribution, with APP accumulating in EEA1 positive structures and the pools of APP in LampI positive late endosomes/lysosomes and the Golgi apparatus diminished (Fig. 4a, b).

These data suggested that APP became trapped in early endosomes or early endosome-derived vesicles upon PIKfyve inhibition with markedly reduced transport to both late endosomes/lysosomes and the Golgi apparatus.

We also studied the protein levels of endogenous APP and APLP2 to test whether they are affected by PIKfyve inhibition. While APLP2 appeared largely unchanged, the overall levels of APP increased strongly and its band pattern in Western blot was altered (Fig. 4c, d). Particularly the 135 kDa form strongly increased in quantity while the approx. 110 kDa form remained largely unchanged. It has previously been shown that human APP expressed from cDNA results in a 135 and 110 kDa form, most likely differing in their degree of glycosylation [49]. Taken together these data show that APP trafficking and APP levels depend on PIKfyve activity.

PIKfyve has two well-established roles in endosomal function: Mediating endosome-to-TGN transport and facilitating endosome/lysosome fusion [16, 19]. APP traffics between the Golgi, plasma membrane and endosomes. APP has previously been shown to undergo retromer-mediated endosome-to-TGN transport [31]. Our data show that APP requires PIKfyve to avoid getting ‘stuck’ in early endosomal-derived vacuoles which is fully compatible with the important role that PIKfyve plays for the sorting of receptors in endosomes [16].

Is PIKfyve function also required in neuronal cells for APP trafficking? We tested this question by studying APP trafficking in SH-SY5Y cells. As in HeLa cells, inhibition of PIKfyve using Apilimod led to strong vacuolation and APP-GFP trapping in vacuoles (Online Resource 8), suggesting that PIKfyve is also required in neuronal cells for APP trafficking.

**Fig. 5:**
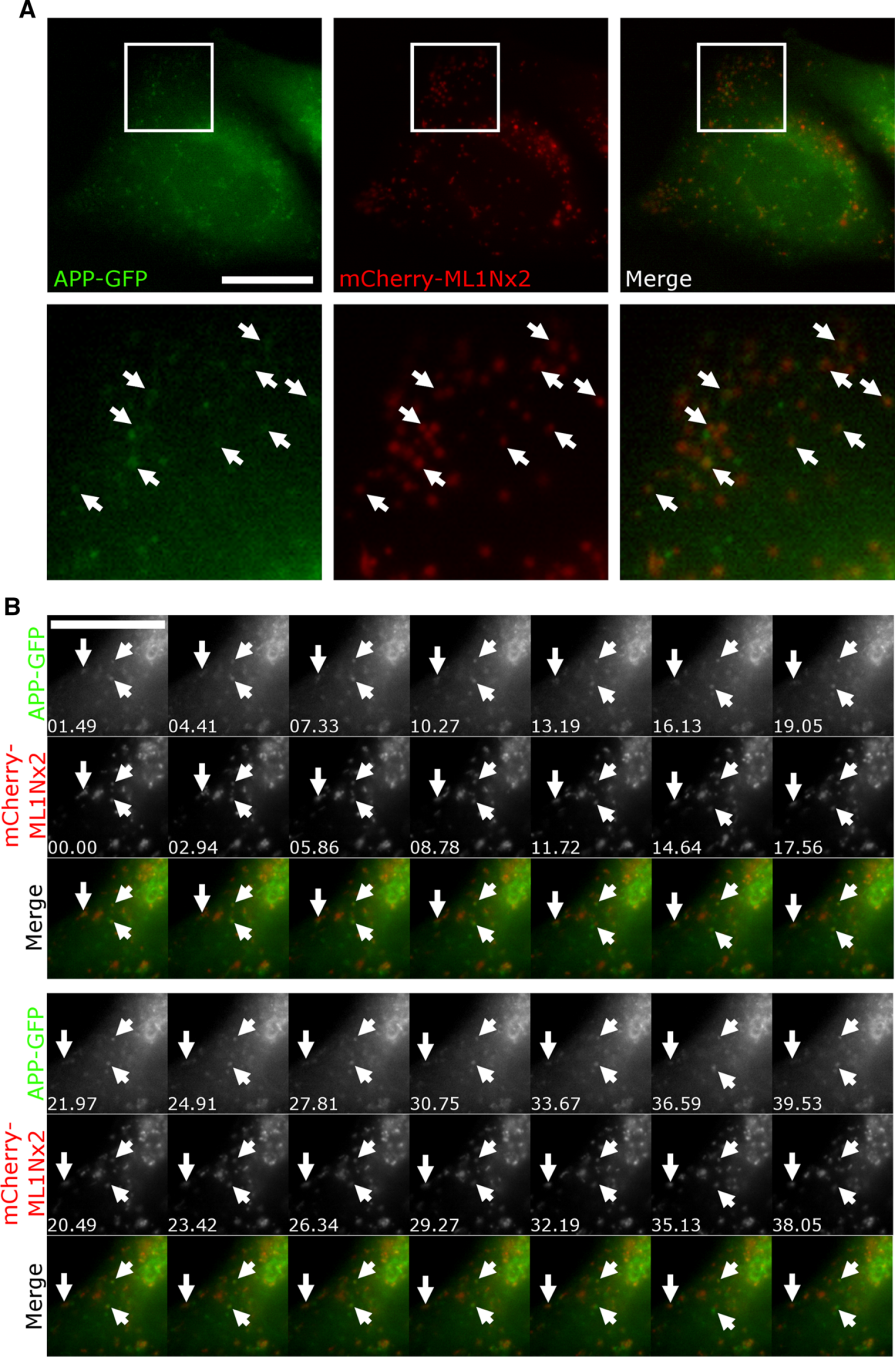
APP co-localises and co-migrates with the PI(3,5)P_2_ marker ML1Nx2. **a** Live cell imaging of APP-GFP and mCherry-ML1Nx2 evidenced colocalisation of both on multiple vesicles throughout the cell (examples are indicated by *arrows* in enlargements). **b** Co-labelling of APP-GFP and mCherry-ML1Nx2 positive vesicles showed that both reside on vesicles that track through the cell (examples are indicated by *arrows*). *Bars* 20 µm

It is conceivable that the endosomal population of APP, by binding to and stimulating PIKfyve can drive local production of PI(3,5)P_2_ and formation of carriers (as suggested by the ML1Nx2 probe in Fig. 2) that may allow APP sorting and escape from endosomes. If this is the case we would expect a fraction of the APP label to co-localise and co-migrate with ML1Nx2. We tested this by co-expressing APP-GFP and mCherry-ML1Nx2 and analysed their behaviour in live cell imaging (Fig. 5a, b). APP-GFP, when expressed at low levels, displayed extensive co-localisation and co-migration with mCherry-ML1Nx2 (Online Resource 9), consistent with the idea that PI(3,5)P_2_ production is required for APP trafficking as suggested by our PIKfyve inhibition experiments.

The analysis of the interaction interface between APP and Vac14 highlighted the significance of the YENPTY motif and the C-terminal, adjacent sequence (eliminated in AICD-Tr.3). By consequence deletion of this sequence is expected to disrupt PIKfyve-dependent APP sorting. However, an important caveat is that the YENPTY motif contains a classical NPxY endocytosis motif. Additionally the C-terminal tyrosine of the YENPTY motif is part of the YKFFE AP-4 binding motif required for APP exit from the Golgi [29]. We created the APP-Tr.3-GFP deletion construct in which the FFEQMQN motif was deleted and analysed its trafficking. Entirely consistent with work by [29] APP-Tr.3-GFP accumulated in the Golgi (Online Resource 10), virtually eliminating endosomal localisation, precluding a closer analysis of PIKfyve-dependent, endosomal sorting.

## Discussion

We have shown that the APP C-terminus binds the Vac14 subunit of the PIKfyve complex, an interaction necessary for the formation of vesicles that are positive for the TPRML-1 derived ML1Nx2 probe. The careful characterisation of this probe by Li et al. suggests that the ML1Nx2 probe is indeed PI(3,5)P_2_ specific. For independent confirmation, we used PIKfyve inhibition and studied the impact on the ML1Nx2 probe. PIKfyve inhibition nearly completely eliminated vesicular localisation of ML1Nx2, also suggesting that ML1Nx2 is a *bona*-*fide* PIKfyve function reporter.

To our knowledge, this is the first mammalian Vac14 binding partner established outside the PIKfyve complex that has been shown to modify PIKfyve function. Previously it was shown that PIKfyve could interact with Rab9 effector p40 and the kinesin adaptor JLP [50, 51].

We found that both APP and AICD overexpression were able to stimulate formation of PI(3,5)P_2_ positive vesicles, suggesting that APP is able to modulate or direct PIKfyve activity. It could be speculated that APP, as a transmembrane protein may provide membrane attachment for the PIKfyve complex. In that case, AICD should act as a competitive inhibitor for PIKfyve complex membrane attachment. AICD overexpression should lead to dissociation of PIKfyve from the membrane and reduce PIKfyve’s ability to form PI(3,5)P_2_. However, we observed that AICD overexpression increased the number of PI(3,5)P_2_ positive vesicles to the same extent as APP overexpression in HeLa cells, and to a slightly lower extent in SH-SY5Y cells. It is also worthwhile noting that PIKfyve possesses the PI(3)P binding FYVE domain which is well established to allow membrane association with endosomes [52]. Both facts argue against a role for APP in providing membrane attachment for the PIKfyve complex and rather argue for a role in modulating its activity.

**Fig. 6:**
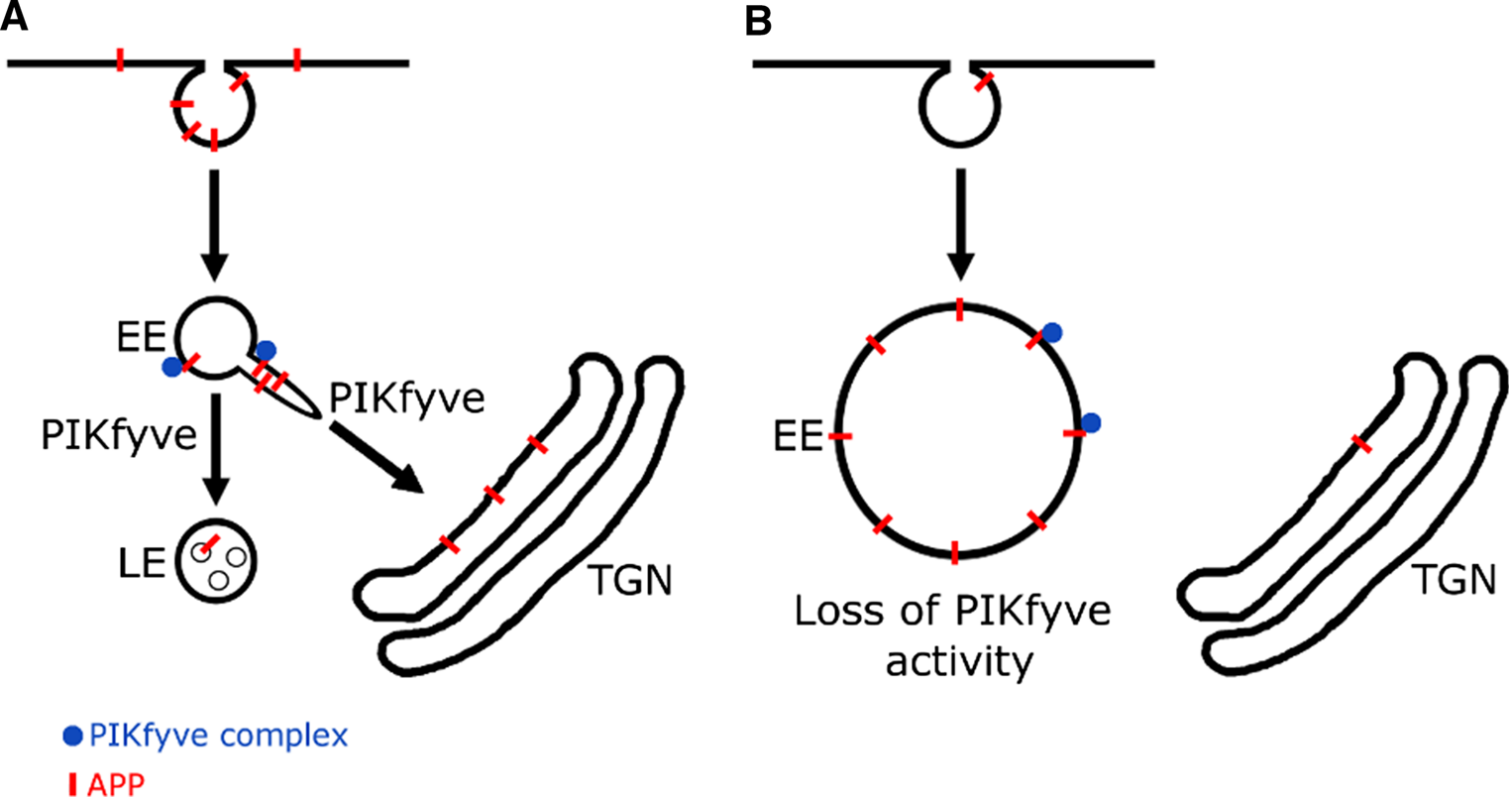
Working model of the APP/PIKfyve interplay. Our data suggest that APP, upon delivery to early endosomes can bind to and stimulate PIKfyve to produce PI(3,5)P_2_ positive vesicles. This may allow APP sorting and escape from endosomes. PIKfyve is necessary for APP sorting from endosomes, as pharmacological inhibition demonstrated that APP remains ‘stuck’ in endosome-derived vacuoles. Our data also suggest that PIKfyve function is required for APP trafficking to LampI positive late endosomes and lysosomes and potentially APP downregulation

We have shown that APP and APLP2 knock-down reduced the number of PI(3,5)P_2_ positive vesicles and increases the susceptibility of cells to form vacuoles, particularly when PIKfyve function is compromised. We have also shown that APP trafficking is dependent on PIKfyve activity in both HeLa and SH-SY5Y cells. From these data we propose a working model in which APP, upon arrival in the early endosome, interacts with the PIKfyve complex by binding Vac14 and triggers the formation of PI(3,5)P_2_ positive vesicles. This allows APP (and potentially other cargoes) to be sorted away from the endosomal system. Pharmacological inhibition of PIKfyve activity suppressed this interplay and led to the trapping of APP in early endosomal, aberrant, vacuolar structures (Fig. 6).

This study revealed an interesting reciprocal relationship between APP and the PIKfyve complex in which APP can bind to the PIKfyve complex, stimulate formation of PI(3,5)P_2_ positive vesicles and in turn regulate its own trafficking. Thus, we have established a novel role for members of the APP gene family as regulators of endosomal phosphoinositide metabolism. This function is certainly shared by APP and APLP2. Whether this is equally true for the brain-specific APLP1 remains to be tested; however, as all three can bind Vac14 of the PIKfyve complex this is likely.

From our data it is clear that the intracellular domain of APP is crucial for its interplay with PIKfyve. Most likely it modulates PIKfyve function by direct binding to the Vac14 subunit of the complex. Vac14 is known to act as a scaffold that brings PIKfyve and Fig4 together [13]. However, how exactly binding of APP to Vac14 may translate into altered PIKfyve activity is unclear in the absence of any structural information. It is possible that APP binds PIKfyve or Fig4 in addition to Vac14. So far we have not been able to express and purify Fig4 or PIKfyve, so we have not been able to test this directly. However, one argument weighs against the idea of an additional binding site for another PIKfyve complex member in the APP intracellular domain: In all interaction experiments in which we studied various AICD mutants (deletion or ‘double point’), binding of PIKfyve to AICD behaved exactly as binding to Vac14, e.g., any mutation abolishing binding to Vac14 also abolished the binding to PIKfyve. This observation does not easily fit with the possibility of an additional binding site in the APP intracellular domain for PIKfyve. However, we cannot currently exclude the possibility.

Another open question is how PIKfyve function enables endosomal sorting. While it is clear that the enzyme plays a pivotal role for endosome-to-TGN transport, the mechanism thereof remains unclear [16]. Answering this question will be challenging as our knowledge of PI(3,5)P_2_ effectors that may contribute to endosomal sorting is extremely limited. However, combining the ML1Nx2 probe and APP as a prospective cargo may provide valuable insights into the formation of PI(3,5)P_2_ vesicles and how PIKfyve function underpins endosomal receptor sorting.

### Implications of the APP/PIKfyve interplay for Alzheimer’s disease

Conclusive genetic as well as biochemical evidence has demonstrated that APP cleavage by beta- and gamma-secretase, producing beta-amyloid on the one hand and destroying APP on the other is a key event in Alzheimer’s disease. Our work has shown that APP biochemically and functionally interacts with the PIKfyve complex. Previous work from the Weisman and Meisler labs has clearly demonstrated that the PIKfyve complex is central to neuronal function and integrity. Compromised PIKfyve function by knock-out or reduced activity of the PIKfyve complex led in all cases to endosomal dysfunction coupled with dramatic neurodegeneration as evidenced in mice and humans [14, 17, 21, 22]. All of the data presented here suggest that APP family members act as PIKfyve activators to support PIKfyve function. This is supported by our recent study in which we showed that the *C. elegans* homologue of APP, APL-1 is genetically linked to PIKfyve and is required for PIKfyve function in the nematode (Balklava et al., in press). This interplay is likely to be perturbed by aberrant APP processing as observed in Alzheimer’s disease. Excessive cleavage of APP is likely to reduce its ability to interact with and stimulate PIKfyve function, thereby compromising endosomal sorting and homeostasis. Disrupted endosomal function (including autophagy) is well established to occur early during Alzheimer’s disease and is thought to contribute to neurodegeneration [53]. By consequence, a perturbed APP/PIKfyve interplay could represent an entirely novel mechanism by which APP cleavage may cause neurodegeneration.

This idea highlights a number of open questions: (1) How does a compromised endo/lysosomal system as caused by loss of PIKfyve function lead to neurodegeneration? (2) Can evidence for PIKfyve dysfunction be obtained in Alzheimer’s disease models or patient samples? (3) Could pharmacological, ectopic activation of PIKfyve reduce/eliminate endosomal dysfunction and prevent neurodegeneration? If so, this would provide an alternative to the largely failed quest for preventing beta-amyloid production or deposition as treatment in Alzheimer’s disease. Answering these questions may provide a completely novel view of the causes of neurodegeneration in Alzheimer’s disease.

### Electronic supplementary material

Below is the link to the electronic supplementary material.

Supplementary material 1 (PDF 3162 kb)

Supplementary material 2 (AVI 21644 kb)

## Abbreviations

AICD: Amyloid precursor protein intracellular domain
APP: Amyloid precursor proteins
APPΔAICD: APP lacking its intracellular domain
BACE1: Beta secretase
BSA: Bovine serum albumin
PBS: Phosphate buffered saline
MBP: Maltose binding protein
PI(3)P: Phosphatidylinositol-3-phosphate
PI(3,5)P_2_: Phosphatidylinositol-3,5-bisphosphate
TGN: Trans-Golgi-network
