# Supplementary material for: APP controls the formation of PI(3,5)P2 vesicles through its binding of the PIKfyve complex
Source: Cell Mol Life Sci. 2015 Jul 28;73(2):393–408. doi: 10.1007/s00018-015-1993-0 (PMC4706845; doi:10.1007/s00018-015-1993-0)
Supplement: Supplementary file 1 — Supplementary material 1 (PDF 3162 kb) [file 18_2015_1993_MOESM1_ESM.pdf]

Online Resources

"APP controls the formation of PI(3,5)P_2 vesicles through its binding of the PIKfyve complex."

Cellular and Molecular Life Sciences

Heather Currinn, Benjamin Guscott, Zita Balklava, Alice Rothnie and Thomas Wassmer*

School of Life and Health Sciences, Aston University, Aston Triangle, Birmingham, B4 7ET, United Kingdom

*) Author for correspondence: t.wassmer@aston.ac.uk

Online Resource 1

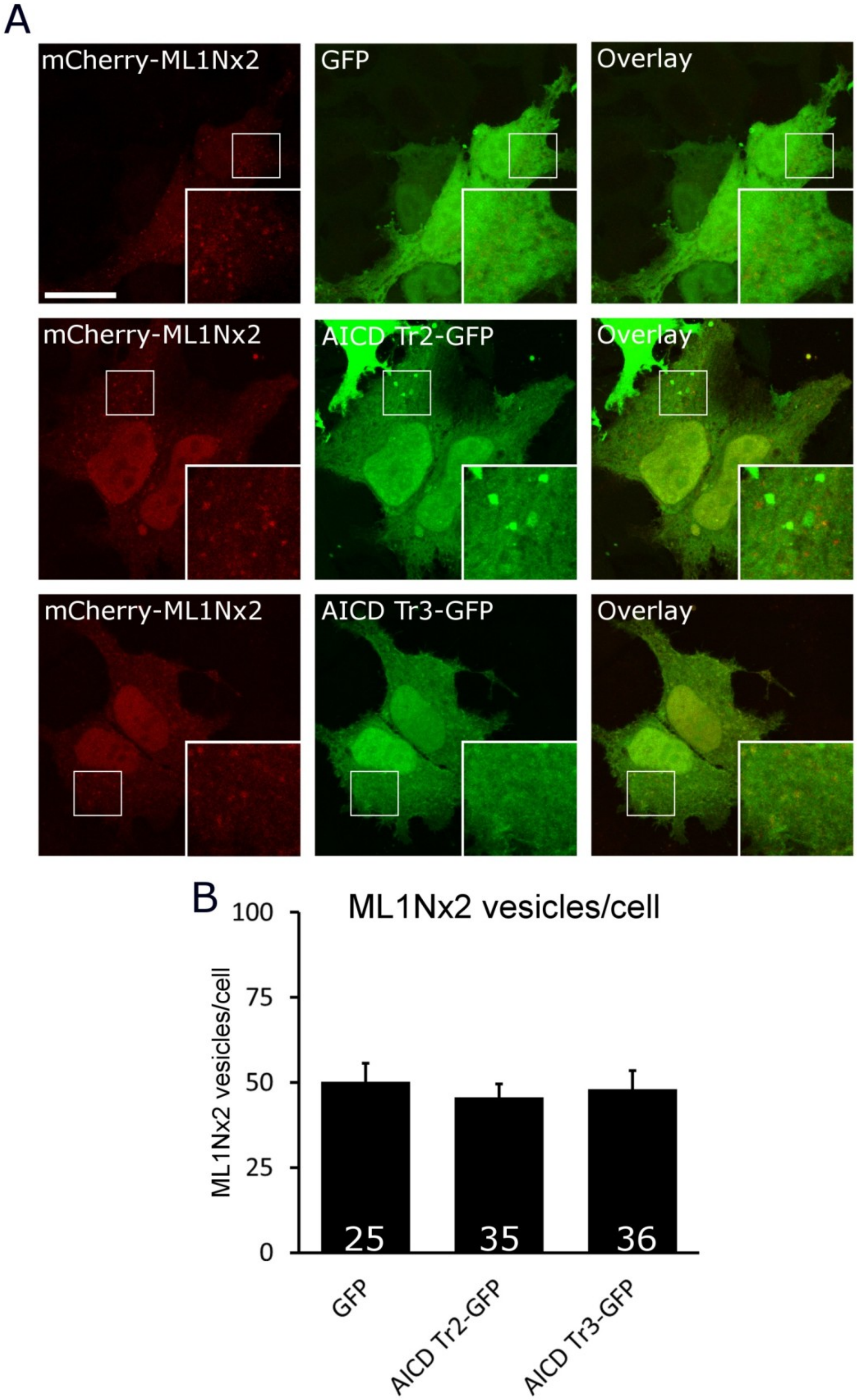

Online Resource 1) Expression of AICD truncations defective in binding the PIKfyve complex fail to stimulate the formation of ML1Nx2 positive vesicles

(A, B) AICD-Tr. 2 and 3 were coexpressed together with mCherry-ML1Nx2. Neither significantly altered the number of mCherry-ML1Nx2 positive vesicles compared to the GFP negative control, confirming that the AICD C-terminus, required for binding the PIKfyve complex is also necessary for stimulating ML1Nx2 positive vesicle formation. (A) Bar, 20um. (B) Error bars are s.e.m., number of total cells analysed in three independent experiments are indicated in each bar. Between 8 and 12 stacks were imaged per experiment. Bar, 20 µm.

Online Resource 2

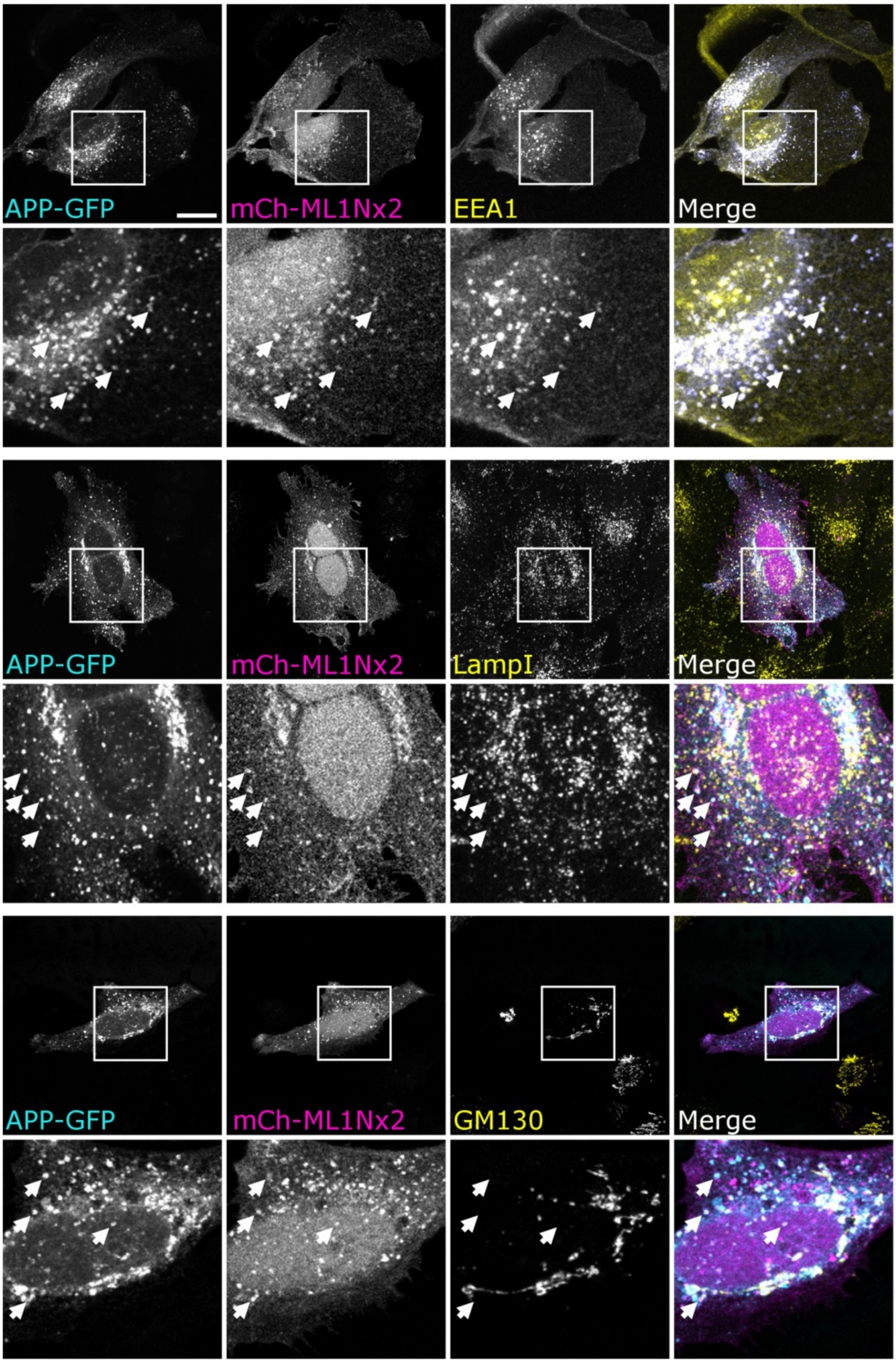

Online Resource 2) Analysis of APP-GFP and mCherry-ML1Nx2 positive vesicles

Co-localisation analysis of vesicles labelled with APP-GFP and mCherry-ML1Nx2 and co-staining with EEA1, LampI or GM130 revealed that vesicles were mainly EEA1 positive, while very limited overlap with either LampI or GM130 could be detected. Arrows indicate the presence of APP-GFP and mCherry-ML1Nx2 positive vesicles and their position relative to the marker. Bar, 20 μm.

Online Resource 3

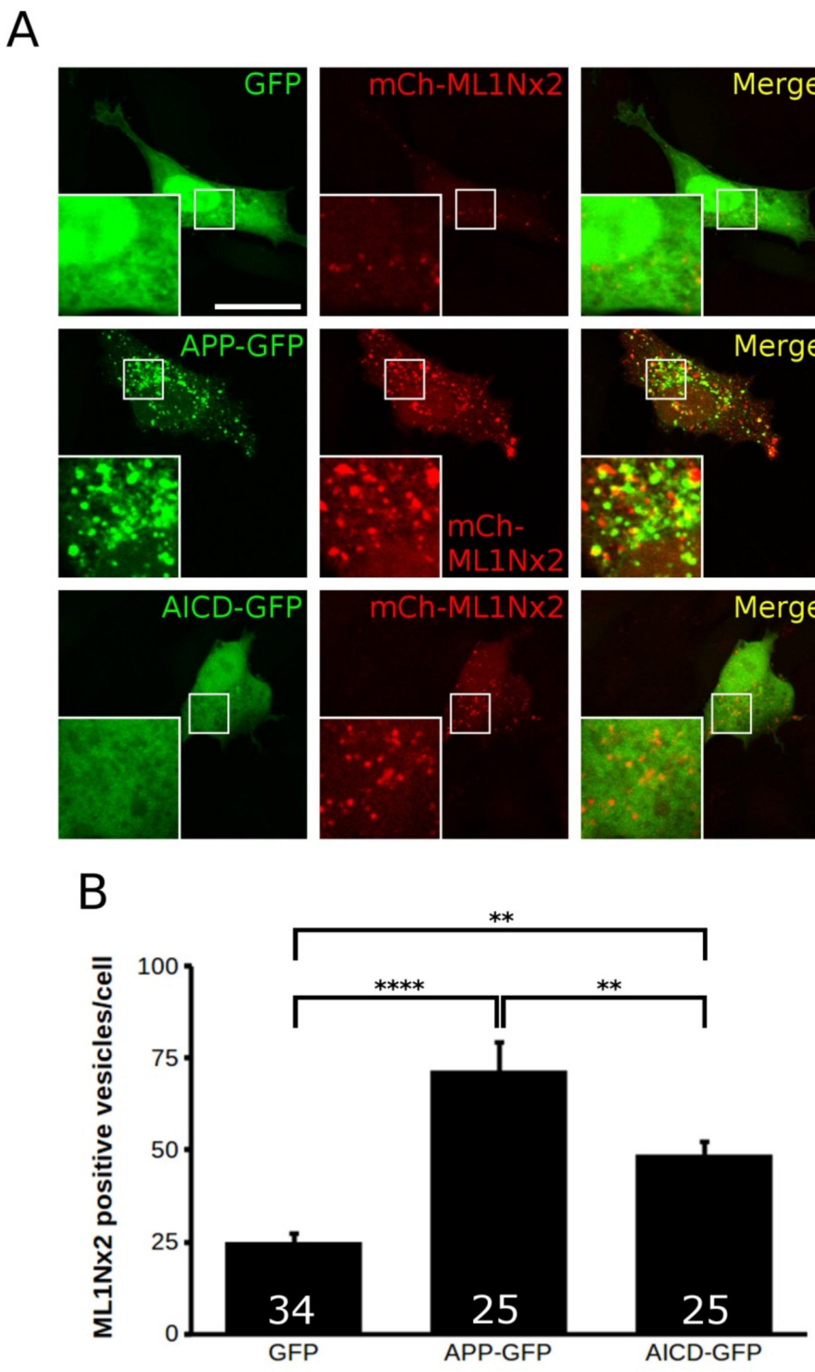

Online Resource 3) Overexpression of APP-GFP or AICD-GFP increases the number of ML1Nx2 positive structures in SH-SY5Y neuroblastoma cells

(A, B) Co-expression of GFP, APP-GFP or AICD-GFP and the mCherry-labelled PI(3,5)P_2 probe ML1Nx2 was used to analyse the impact of APP and AICD expression on ML1Nx2 positive vesicles compared to GFP as negative control. (B) Automated quantification of the number of mCherry-ML1Nx2 structures using image segmentation (MosaicSuite in ImageJ [40]). APP and AICD expression both significantly increased the number of ML1Nx2 positive structures. Interestingly, APP expression seemed more effective than AICD in driving formation ML1Nx2 positive vesicles in SH-SY5Y cells. Significant differences (ANOVA test followed by Tukey's post-hoc analysis with $\alpha=0.05$) are indicated. **** represents $p\leq0.0001$, ** $p\leq0.01$. Total number of cells pooled from three independent experiments (8-12 stacks per experiment) indicated in the graph.

Online Resource 4

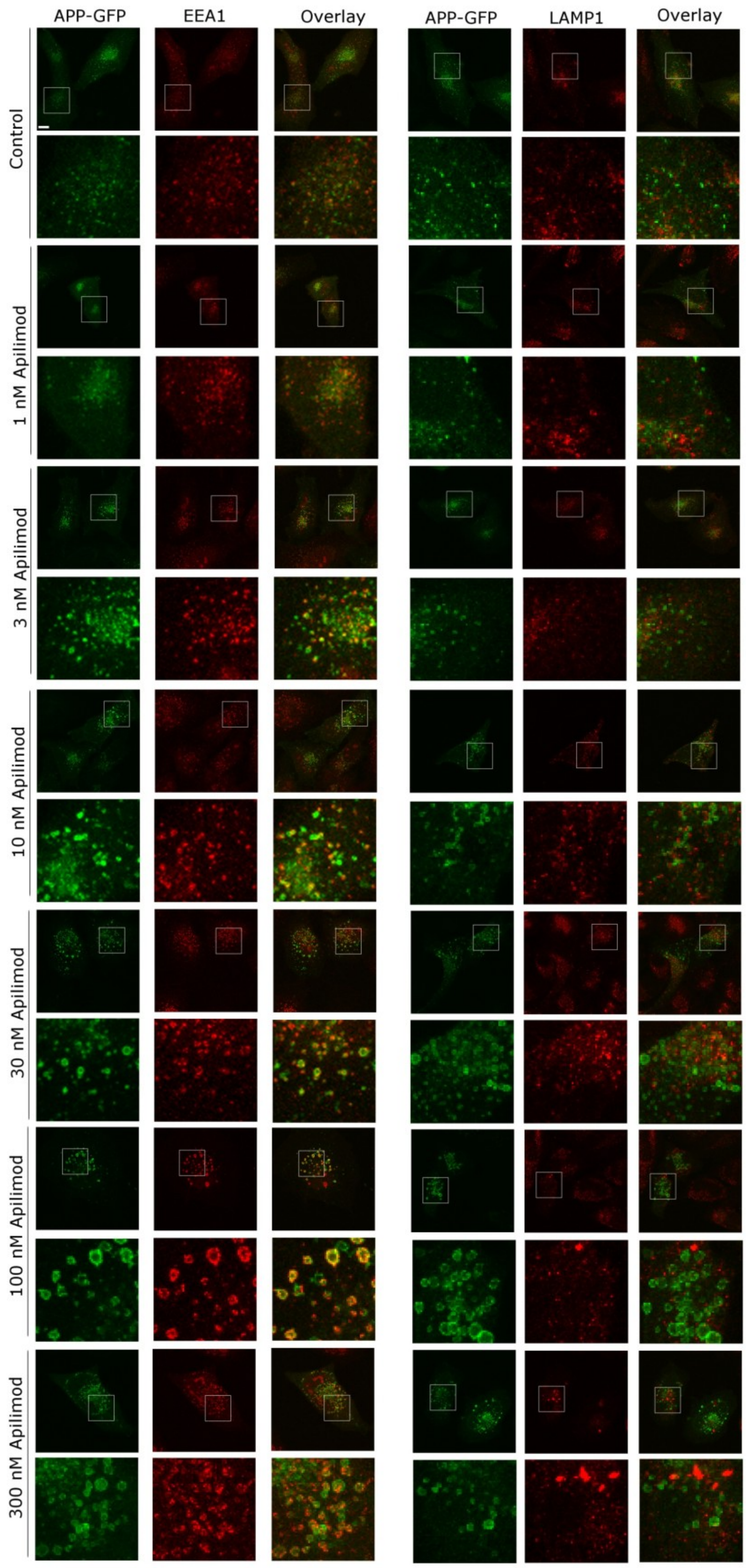

Online Resource 4) Concentration dependence of APP-GFP in endosomes upon PIKfyve inhibition using Apilimod in HeLa cells

PIKfyve inhibition using the indicated concentrations of Apilimod for 4h. Accumulation of APP-GFP in vacuolar structures positive for EEA1 is detectable at concentrations between 10 and 300 nM. At 3 nM APP-GFP vesicles appear enlarged compared to the negative control without any inhibitor. No obvious effect on APP-GFP accumulation in vacuoles was detected using 1 nM Apilimod (n=4). Bar, 10 µm.

Online Resource 5

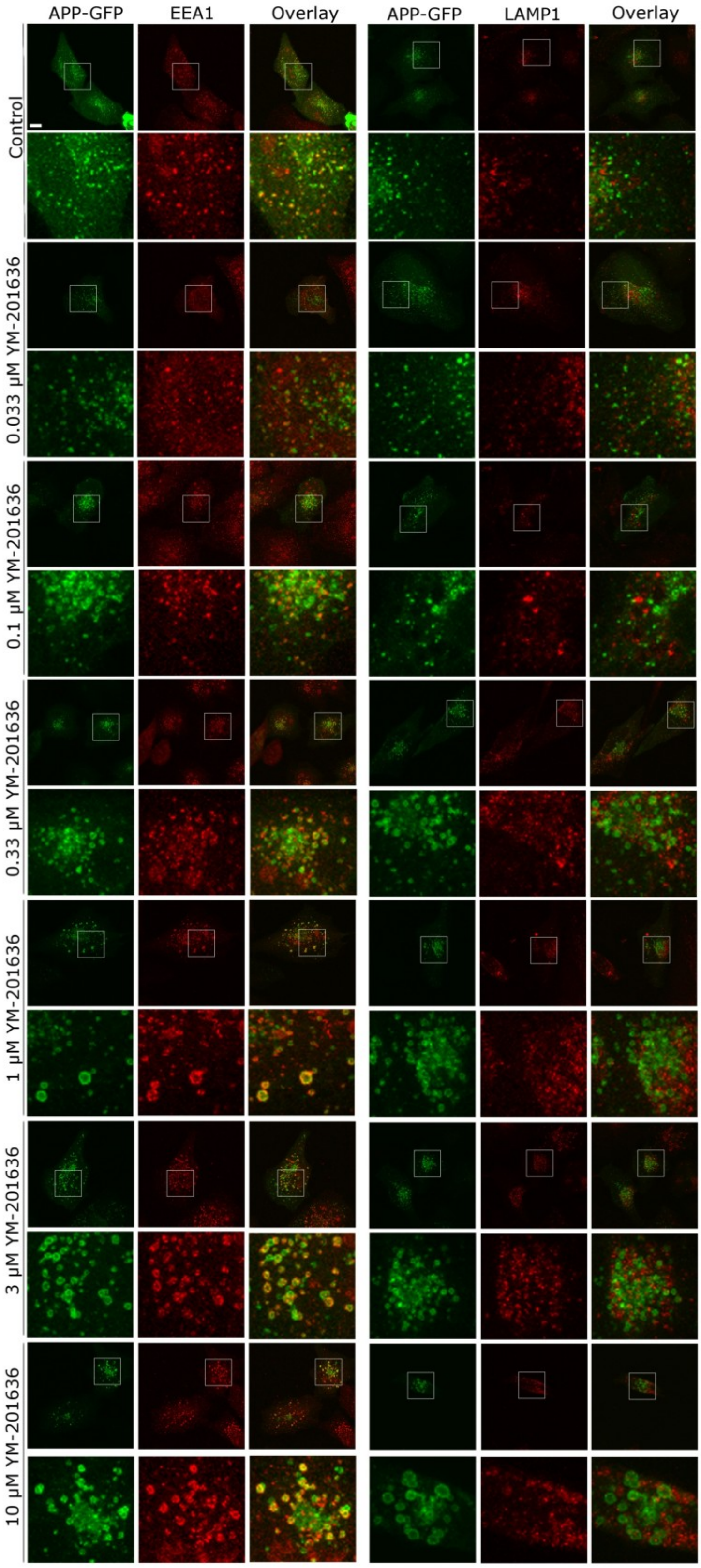

Online Resource 5) Concentration dependence of APP-GFP accumulation in early endosomes upon PIKfyve inhibition using YM201636 in HeLa cells

PIKfyve inhibition using the indicated concentrations of YM201636 for 4 h led to accumulation of APP-GFP in vacuolar structures positive for EEA1 at concentrations between 330 nM and 10 µM. At 100 nM some small, APP-GFP positive vacuoles were detectable. At 100 nM APP-GFP positive vesicles that did not show clear vacuolar morphology (with a discernible ring) appear enlarged compared to APP-GFP vesicles in the negative control. No obvious effect was detected at the lowest YM201636 concentration tested, 33 nM. APP-GFP accumulation showed concentration dependency on the PIKfyve inhibitor YM201636 as shown for Apilimod (Online Resource 4) (n=4). Bar, 10 µm.

Online Resource 6

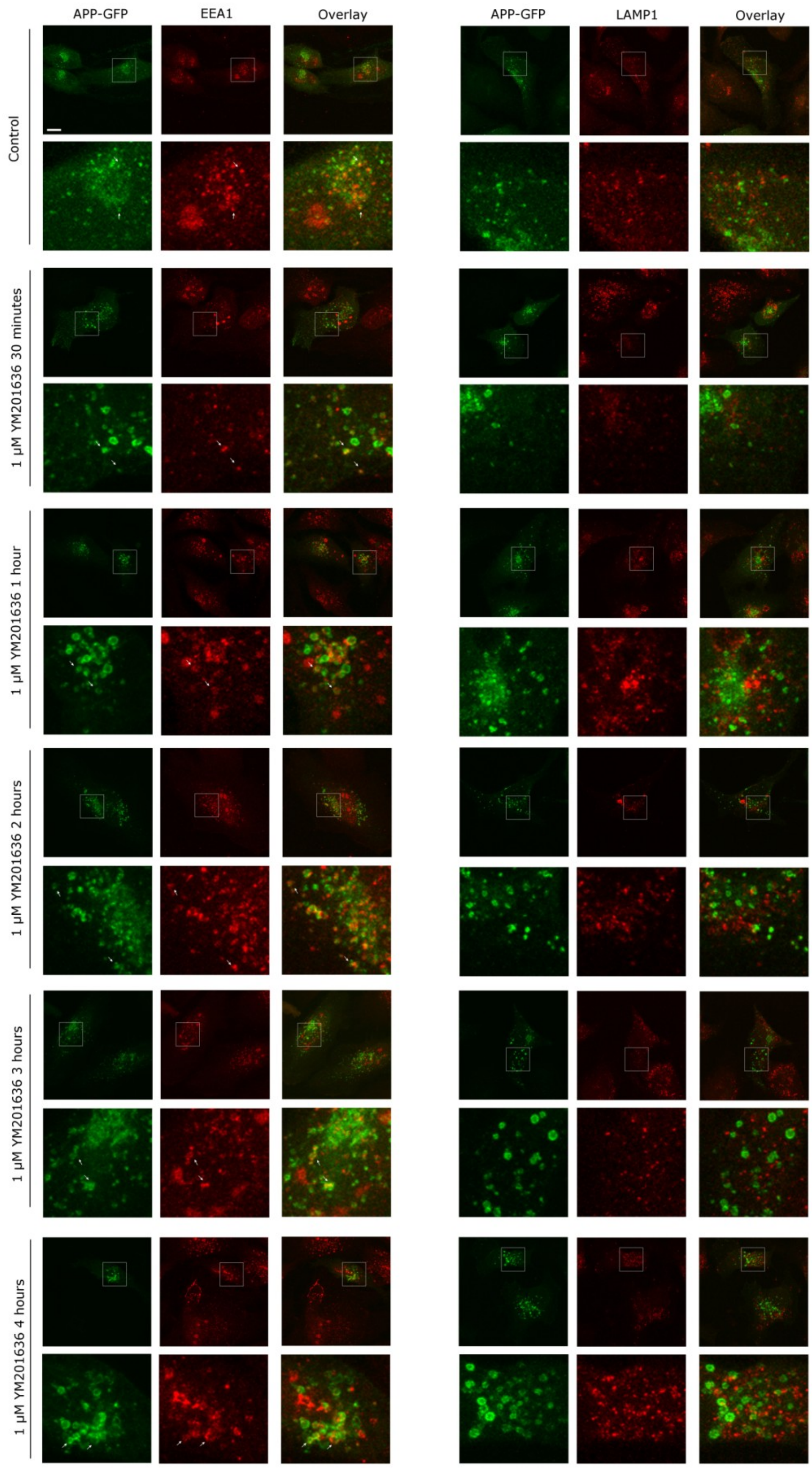

Online Resource 6) Time dependence of endosomal APP-GFP vacuolation upon PIKfyve inhibition using YM201636

Enlarged, vacuolar APP-GFP positive structures could be detected as early as 30 min using 1 μM YM201636. Their number and size increased significantly over the next three and a half hours (n=4). Bar, 10 μm.

Online Resource 7

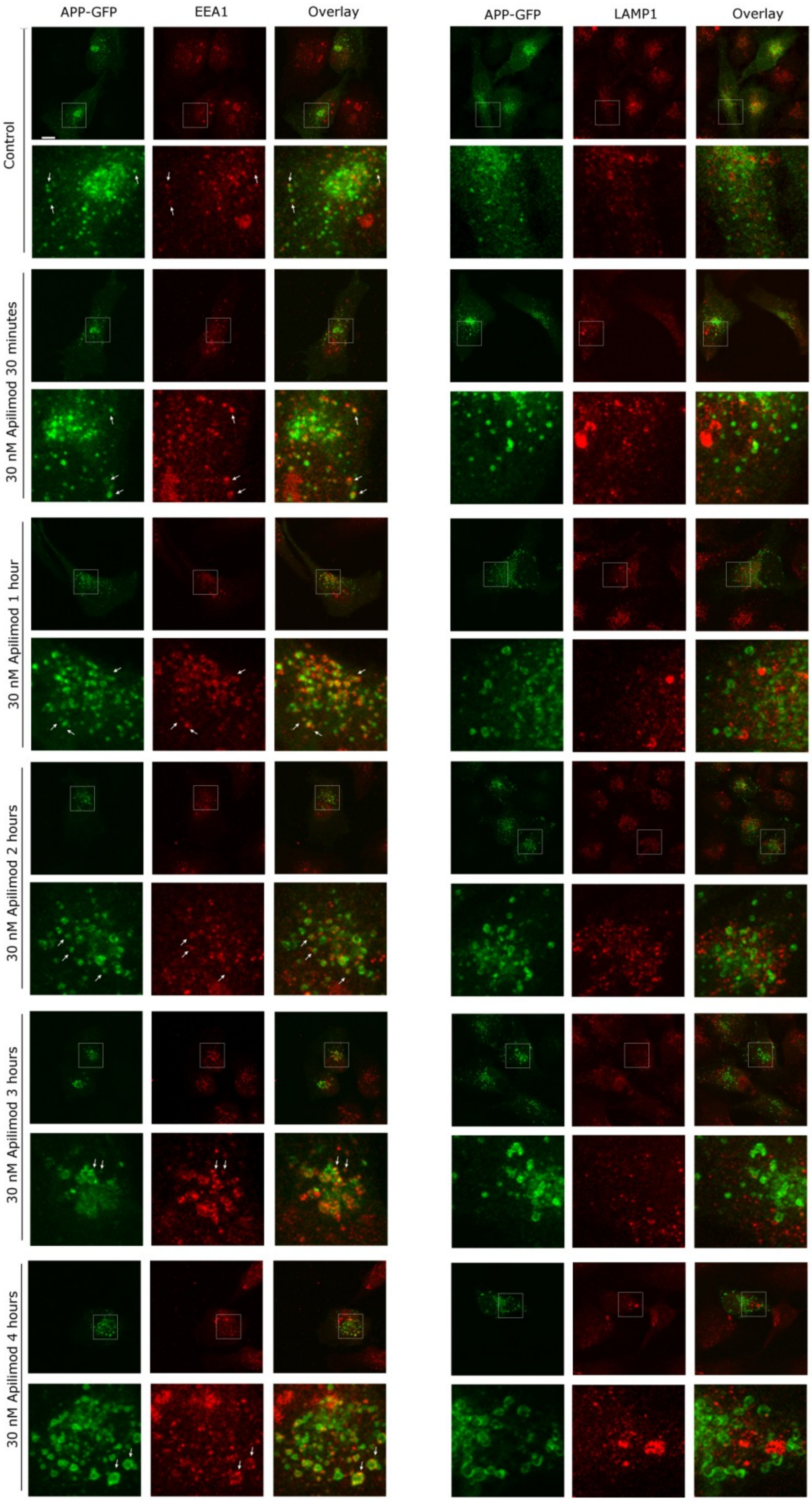

Online Resource 7) Time dependence of endosomal APP-GFP vacuolation upon PIKfyve inhibition using Apilimod

Enlarged APP-GFP positive structures could be detected as early as 30 min. They increased in size and become clearly vacuolar after 1 h. As with YM201636 their number and size progressively increased (n=4). Bar, 10 µm.

Online Resource 8

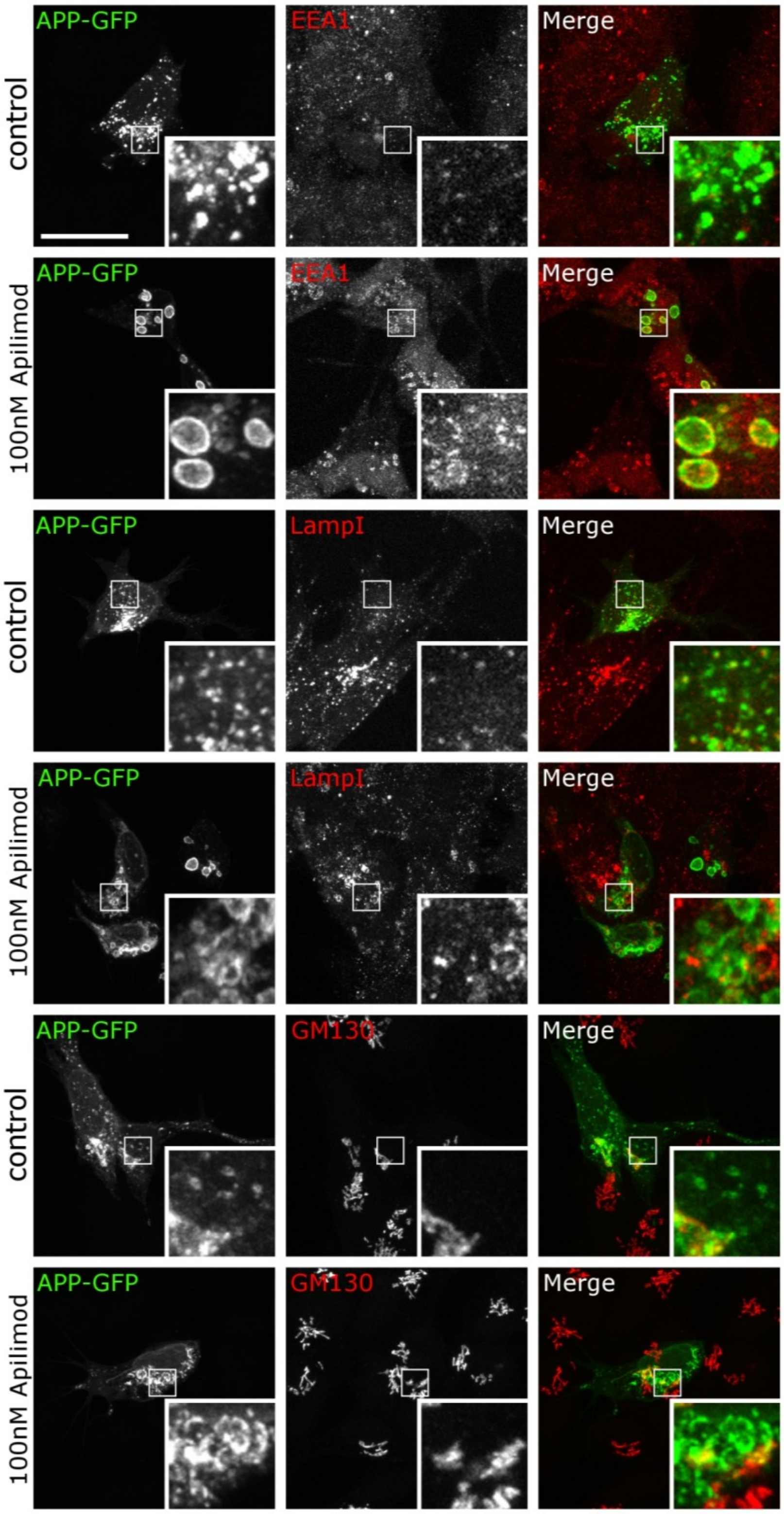

Online Resource 8) APP trafficking depends on PIKfyve in SH-SY5Y neuroblastoma cells

PIKfyve inhibition using Apilimod (100 nM, 4 h) led to extensive vacuolation positive for APP-GFP, similar to the phenotype observed in HeLa cells. Bar, 20 μm.

Online Resource 9) APP-GFP expressed at low levels can be observed to co-localises and co-migrate with mCherry-ML1Nx2 in vesicles throughout the cytoplasm

Left panel: Merge between APP-GFP (green) and mCherry-ML1Nx2 (red). Middle panel: APP-GFP, Right panel: mCherry-ML1Nx2. Note that the green and the red channel were collected with a slight delay due to the time required for changing the filter. Acquisition times are indicated.

Online Resource 10

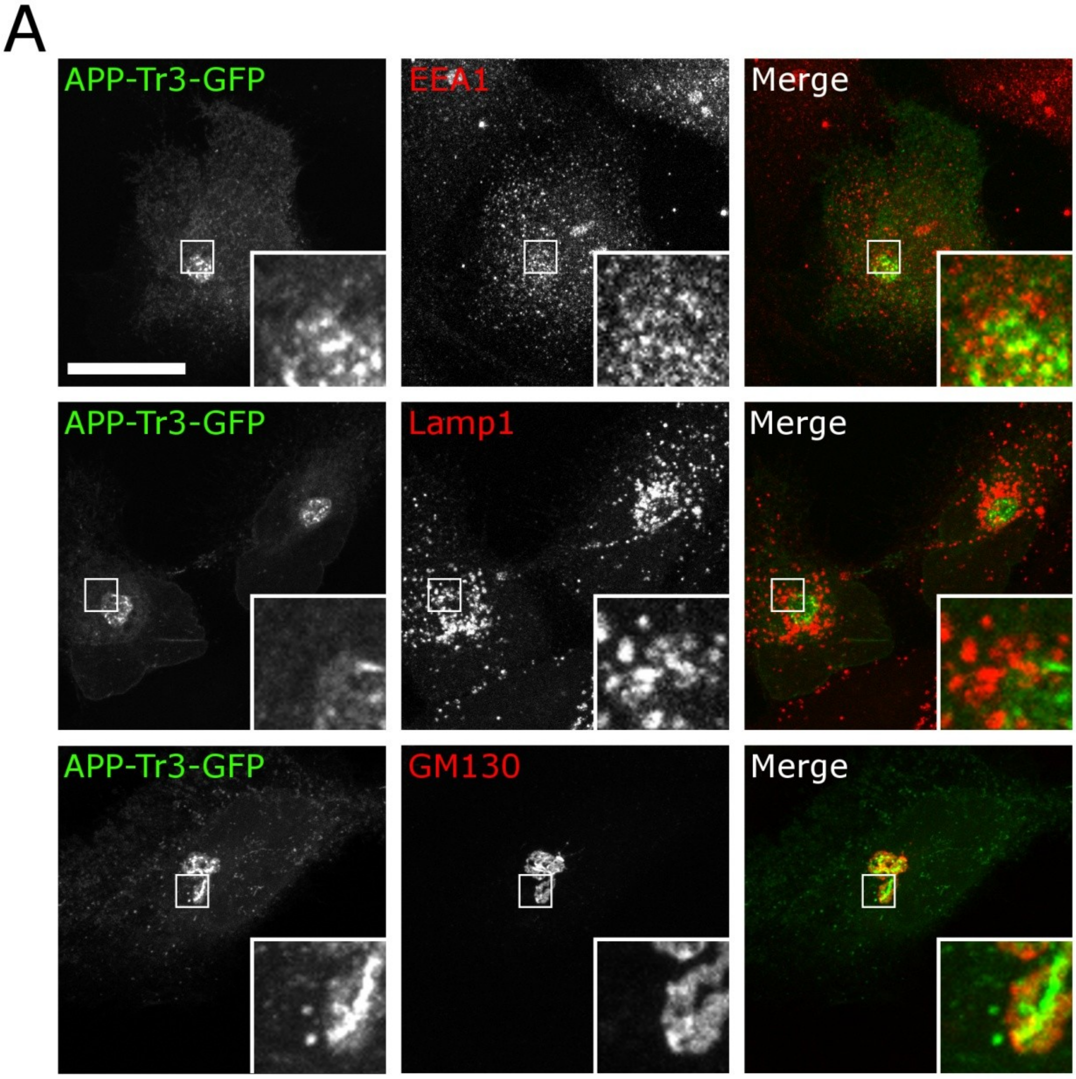

B

AICD KKKQYTSIHHGVVEVDAAVTPEERHLSKMQQNGYENPTYKFFEQMQN*
AICD-Tr3 KKKQYTSIHHGVVEVDAAVTPEERHLSKMQQNGYENPTYK
AICD-Tr2 KKKQYTSIHHGVVEVDAAVTP
AICD-Tr1 KKKQYTSIHH

Online Resource 10) Introduction of Tr.3 into APP-GFP disrupts its trafficking

(A) Expressed APP-Tr.3-GFP was found to mainly localise to the Golgi apparatus in HeLa cells. Note the marked difference in distribution of APP-Tr.3-GFP and APP-GFP (Fig. 4). In APP-Tr.3-GFP expressing cells vesicular staining is largely absent from the cell periphery and most of the label is concentrated in the Golgi area (indicated by GM130). This is consistent with a disruption of APP's AP-4 binding motif (indicated in blue in (B)) in the

APP-Tr.3 mutant. AP-4 binding to APP is required for APP export from the trans-Golgi-network and its trafficking to the endosomal system [29]. (B) Sequence of AICD and AICD-Tr1-3. Disruption of amino acids shown in red disrupted the interaction with the PIKfyve complex (compare Fig. 1). Blue letters indicate the AP-4 binding motif of APP established by [29]. Unfortunately, APP's binding site of the PIKfyve complex overlaps with the AP-4 targeting motif. By consequence it was not possible to study any endosomal APP trafficking defect of APP-TR.3-GFP as this mutant protein remained 'stuck' in the Golgi. Bar, 20 µm.
